# Comprehensive investigating of mismatch repair genes (MMR) polymorphisms in participants with chronic hepatitis B virus infection

**DOI:** 10.3389/fgene.2023.1077297

**Published:** 2023-02-01

**Authors:** Ning Ma, Yucheng Sun, Yanan Kong, Yiyao Jin, Fengxue Yu, Lianfeng Liu, Lei Yang, Wenxuan Liu, Xia Gao, Dianwu Liu, Xiaolin Zhang, Lu Li

**Affiliations:** ^1^ Department of Social Medicine and HealthCare Management, School of Public Health, Hebei Medical University, Hebei Key Laboratory of Environment and Human Health, Shijiazhuang, China; ^2^ Department of Epidemiology and Statistics, School of Public Health, Hebei Medical University, Hebei Key Laboratory of Environment and Human Health, Shijiazhuang, China; ^3^ Division of gastroenterology, The Second Hospital of Hebei Medical University, The Hebei Key Laboratory of Gastroenterology, Shijiazhuang, China; ^4^ Department of Pediatrics, Shijiazhuang Maternal and Child Health Hospital, Shijiazhuang, China

**Keywords:** HBV, MMR, SNPs, haplotype, interaction

## Abstract

**Background and aim:** In this study, we focused on the relationship between single nucleotide polymorphisms in MMR genes and the occurrence and development of HBV infection.

**Materials and methods:** A total of 3,128 participants were divided into five groups: negative control group (NeC), spontaneous clearance group (SC), chronic hepatitis B group (CHB), liver cirrhosis group (LC) and hepatocellular carcinoma group (HCC), CHB, liver cirrhosis and hepatocellular carcinoma constitute HLD. We conducted three case-control studies: NeC (840 cases) vs. HLD (1792 cases), SC (486 cases) vs. HLD (1792 cases) and CHB + LC (1,371 cases) vs. HCC (421 cases). 11 polymorphic loci in *MLH1, MLH3, MSH5, PMS1* and *PMS2* were involved in genotyping by Sequenom MassArray. The SNPStats performed Hardy-Weinberg equilibrium test. Linkage disequilibrium patterns were visualized using Haploview4.2. The GMDR (v0.9) was conducted to generalized multifactor dimension reduction analysis. The correlation, multiplicative interaction and additive interaction analyses were calculated by Logistic Regression through SPSS21.0. Matrix and programmed excel were also involved in the calculation of additive interaction.

**Results:** In NeC vs. HLD group, *MSH5*-rs1150793(G) was a risk base to HBV susceptibility (nominal *p* = 0.002, OR = 1.346). We found multiplicative interaction between *MLH1*-rs1540354 (AA + AT) and *PMS1*-rs1233255 (AA) (nominal *p* = 0.024, OR = 1.240). There was additive interaction between *PMS1*-rs1233255 (AA) and *PMS1*-rs256554(CA + CC). In SC vs. HLD group, *MLH1*-rs1540354 (TT) was a risk genotype (nominal *p* < 0.05, OR>1). Through haplotype analysis, we found the linkage disequilibrium of three loci in *MLH1*. The results of GMDR showed the optimal five-locus model about the spontaneous clearance of HBV. In CHB + LC vs. HCC group, *PMS2*-rs12112229(A) was related to the cancerization of liver.

**Conclusion:** We found rs1150793(G), rs1540354(T) and rs12112229(A) were significantly related to HBV susceptibility, spontaneous clearance of HBV and cancerization after infection, respectively.

## Introduction

Hepatitis B virus (HBV) is the pathogen causing Hepatitis B. The outcomes of HBV infection contain spontaneous HBV clearance and chronic HBV infection. The latter can be further classified into asymptomatic carrier status and chronic hepatitis B (CHB), which could progress to liver cirrhosis (LC) and hepatocellular carcinoma (HCC). It is estimated that 8%–20% of untreated individuals with CHB will develop into cirrhosis within 5 years (Terrault et al., 2016), and 2%–8% of those with cirrhosis develop into HCC in a year (Yim and Lok, 2006; Bruix et al., 2011; Hung et al., 2017). HBV infection has become a serious health problem globally with about 248 million chronically infected individuals (Chang and Nguyen, 2017). Therefore, it is necessary to enhance the study of the mechanisms affecting the occurrence and development of HBV, and provide clinical evidence for the prevention and prognosis evaluation of HBV infection.

The clinical outcome of HBV infection relies on viral, host and environmental factors. Recently, lots of studies revealed many hosts genetic factors played crucial roles in hereditary susceptibility and clinical outcomes of HBV infection. For instance, genetic variations in HLA class II locus were associated with the susceptibility to persistent HBV infection (Matsuura et al., 2016). Besides, m6A modifications of HBV RNA could be one of the leading arms in the immune escape of the virus, resulting in viral persistence and chronicity (Wills and Mead, 2015). Gene polymorphism in the population is the basis of this genetic susceptibility, and it is a common phenomenon in biological populations. Fundamentally speaking, it is caused by gene variation. And it occurs frequently in regions without protein coding function or important regulatory function in gene sequence. Based on the attention and research sequence, human gene polymorphism can be divided into three categories: DNA fragment length polymorphism, DNA repeat sequence polymorphism and single nucleotide polymorphisms (SNPs). Among them, SNPs refer to a base substitution, the most common form of polymorphism, accounting for more than 80% of the already known polymorphism.

SNPs are important genetic biomarker in humans, a number of studies have shown that SNPs play an important role in the occurrence and development of HBV infection. For example, SNPs in the interleukin 28B gene (*IL28B*) can influence the course of treated and untreated HBV infection as well as spontaneous clearance of HCV (Hashemi et al., 2019; Qin et al., 2019; Vergori et al., 2020). In 2010, FDA recommended that *IL28B* genotype could be used as one of the routine evaluation indicators in the clinical trials of new drugs for chronic hepatitis C. Besides, the *ALDH2∗2* polymorphism (rs671) can regulate the inflammatory response caused by acetaldehyde (Zhang and Fu, 2021). And Ganesan found that acetaldehyde decreased the expression of hepatocyte cell surface HBV peptide-MHC class I complexes (Ganesan et al., 2019). This inhibited CTL activation, leading to persistent HBV infection.

In 1993, Fishel found a gene capable of repairing DNA damage in Escherichia coli, which is mismatch repair gene (Fishel et al., 1993). The function of MMR gene is to eliminate base mismatch and insertion/deletion spike ring formation during DNA replication to ensure high fidelity of genetic information. Identifying single-strand gaps in the nascent chain, rather than methylated adenines, makes it possible to excise only the wrong nucleotides in the daughter chain. Based on these functions, the association between the MMR gene and various diseases has been reported. Nowadays, the MMR gene family mainly includes *MLH1*, *MSH2*, *MLH3*, *MSH3*, *MSH5*, *MSH6*, *PMS1* and *PMS2*.

Tan S K showed that in 453 patients with liver cancer, *MLH1* gene polymorphism may have gene-environment interaction with HBV infection and family tumor history after adjusting factors such as age and gender, increasing the risk of liver cancer (Tan et al., 2014). In addition, because *MLH3* participated in multiple biological processes such as DNA repair, microsatellite stability and carcinogenesis (Chen et al., 2005). It was verified that the AA genotype of *MLH3*-rs175080 increases the risk of primary hepatocellular carcinoma for the Han population of northern China (Liu et al., 2015). Chen also found that *MLH3* deficiency has significant consequences for DNA-damage response, gastrointestinal and extragastrointestinal, mutation avoidance and life span (Chen et al., 2005). In addition, Fang proposed that functional loss mutations within *PMS1* increases the frequency of mutations and promote tumorigenesis (Chong et al., 2013; Fujikura et al., 2018). The loss of MLH1/PMS2 complexes and (or) MSH2/MSH6 complexes expression is the most common pattern in MMR deficiency tumors (Salem et al., 2020).

Thus, this study was performed to identify the potential relationships between the SNPs of five genes (*MLH1*, *MLH3*, *MSH5*, *PMS1* and *PMS2*) and HBV infection, which will provide important references for the occurrence, development and deterioration of hepatitis B. We first considered the correlation between all selected loci as well as some environmental factors and the occurrence and development of HBV infection. In addition, linkage disequilibrium (LD) was observed at several loci on the same chromosome in our study. Because the genetic information provided by haplotype is more accurate and more consistent with the genetic characteristics of polygenic diseases than that provided by a single allele. Haplotype analysis has become a very effective method to explore the association between genomes and diseases, and has gradually become an effective tool to study the risk factors of complex diseases. Gene-gene and gene-environment interaction are significant in the pathogenesis of ‘complex trait disease’ or common chronic diseases. The profound exploration of gene-gene interaction will be productive to grasp the causes of population susceptibility discrepancies, and then have a comprehensive understanding of the relationship between genes and diseases. By applying it to disease prevention, we can formulate more useful counter-measures according to individual combined genotypes (Lee et al., 2016).

In view of the above, based on the candidate gene strategy, eleven loci in *MLH1, MLH3, MSH5, PMS1* and *PMS2* were selected into the study. This project consisted of three case-control studies: the genetic factors related to HBV susceptibility were studied with NeC group as control group and HLD group as case group; taking SC group as the control group and HLD group as the case group aimed at investigating the genetic factors affecting the ability of HBV spontaneous clearance; taking CHB + LC group as the control group and HCC group as the case group to evaluate the relationship between SNPs and cancerization after HBV infection. And we studied the relationship between the genetic variation of the selected gene loci and the occurrence and development of HBV infection by performing correlation, haplotype and interaction analyses, which could make the results more accurate and reliable.

## Materials and methods

### Study participants

Three case-control studies were conducted to research the occurrence and development of HBV infection. In the regression analysis, up to 40 independent variables including 36 pairs of loci interaction and other four factors (age, gender, the history of smoking and drinking) were included in Logistic Regression equation. Therefore, there were at least 400 samples in a single group. A total of 3,128 objects meeting the inclusion criteria were recruited from the First, Second and Fourth Hospitals of Hebei Medical University and the Fifth Hospital of Shijiazhuang City. Then they were divided into five groups, including negative control group (NeC: 840 cases), spontaneous clearance group (SC: 496 cases), chronic hepatitis B group (CHB: 691 cases), liver cirrhosis group (LC: 680 cases) and HBV-related hepatocellular carcinoma group (HCC: 421 cases). CHB, LC and HCC were collectively referred to as HBV-induced liver diseases group (HLD).

The NeC group was defined as a group that had never been infected with HBV before, and all serological markers of hepatitis B were negative or only anti-HBs was positive. Blood routine and biochemical indexes of liver function were normal. Liver imaging examination showed no organic liver lesions. The SC group needed to meet three conditions: (ⅰ) The combined positive of anti-HBs and anti-HBc in serum; (ⅱ) HBV DNA, HBsAg and HBeAg were all negative; (ⅲ) Blood routine and biochemical indexes were within the normal range. The inclusion criteria of CHB accorded with *China’s 2019 edition of Guidelines for Prevention and Treatment of Chronic Hepatitis B*. The inclusion criteria of LC and the inclusion criteria of HCC met *China’s 2019 Guidelines for Diagnosis and Treatment of Liver Cirrhosis* and *China’s 2019 edition of Guidelines for Diagnosis and Treatment of Primary Liver Cancer* respectively.

Some exclusion criteria were also set, such as the patients with acute hepatitis B and secondary HBV-HCC. Patients with liver injury caused by non-viral factors such as alcohol and drugs or hepatitis caused by other virus were also excluded.

Han population in northern China who met the above diagnostic criteria were included. We collected their complete clinical information and general information including age, gender, career, marital status, family history, medical history, the history of smoking and drinking and so on by questionnaires and clinical records. All the participants signed the informed consent and the Ethics Committee of Hebei Medical University approved this study.

### The selection of candidate genes and SNPs

GWAS has identified thousands of SNPs related to diseases, and most of them are located in non-coding regions (Suppiah et al., 2009; Wang et al., 2022). Compared to the transcription function of gene coding region, non-coding region has more regulatory functions (Beermann et al., 2016). For example, the single base change of transcription factor binding site (TFBS) may affect the whole process of gene expression. Expression quantitative trait locus (eQTL) analysis is an important tool to study the relationship between genome variation and transcriptome expression level. Therefore, this study selected 11 candidate SNPs with different functions such as enhancer, TFBS, ESE (Exonic Splicing Enhancer), ESS (Exonic Splicing Silencer) and eQTLs through literature search (Langeberg et al., 2010; Bonadona et al., 2011; Gao et al., 2011; Zhu et al., 2017; Salem et al., 2020; Guan et al., 2021) and database search. In the process of selecting SNPs, PubMed database (https://www.ncbi.nlm.nih.gov/pubmed/) was used to search ‘MMR and cancer\tumor’. The specific physical location and MAF of SNPs in the Han population in northern China were searched through the databases UCSC (http://genome.ucsc.edu/) and Ensembl (http://asia.ensembl.org/index.html). GWAS4D, SEdb (http://sea.edbc.org), eQTLGen Consortium (https://eqtlgen.org/) and SNPinfo Web Server (SNPinfo Web Server (nih.gov)) were used to predict the function of SNPs. Finally, 11 polymorphic loci located in five genes met our inclusion criteria. The details are shown in Supplementary Table S1. The MAF of each locus is greater than 0.05 in the Han population in northern China.

### DNA extraction and SNP genotyping

2 mL of anticoagulated peripheral venous blood was collected by ethylenediamine tetra-acetic acid (EDTA) from every participant. Then the blood samples were stored at 4°C. Genomic DNA Purification Kits (Promega, the US) were used for DNA extraction. The extracted DNA was frozen and stored at −80°C. The Sequenom MassArray system was employed for genotyping. Primer sequences for the 11 SNPs used for MassARRAY genotyping were shown in Supplementary Table S2. Polymerase chain reaction (PCR) started with a 15-min initial denaturation at 94°C. According to the process of denaturation-annealing-extension, amplification for 45 cycles was carried out, with denaturation at 94°C for 20 s, annealing at 56°C for 30 s and extension at 72°C for 1 min. The final extension was performed at 72°C for 3 min and then the product was cooled to 4°C. A reverse-phase absorption elution was used to desalt the samples and the MassARRAY Typer 4.0.5 Software was used for genotyping analysis. For quality control, 1% of each plate was reserved for template positive and negative controls, and the analysis of data was repeated for selected 10% of the samples randomly.

### Statistical analysis

The collected data was double entered into EpiData3.1 after invalid questionnaires were removed. Then, we classified and coded the answers of the questionnaire, cleaned the data and checked the errors logically. The SNPStats performed Hardy-Weinberg (H-W) equilibrium test on genotypic frequencies at 11 loci in individuals of the total population. SPSS21.0 (SPSS Corporation, Chicago, IL, United States of America), Haploview4.2 (Copyright c) 2003–2006 Broad Institute of MIT and Harvard, United States) and GMDR (v0.9) were conducted for statistical analysis. Pearson chi-square test and fisher-exact test were used to compare the rate or composition ratio of two or more samples. The aim of unconditional Logistic Regression analysis was to study the relationship between SNPs and disease occurrence and development. Haploview4.2 was used to analyze the association between haplotypes and diseases. GMDR (v0.9) was conducted to carry out the generalized multifactor dimension reduction analysis. The gene-gene multiplicative interaction was evaluated by Logistic Regression model. The additive interaction analysis was carried out jointly by SPSS21.0 and Excel (2019). Firstly, calculate B, *P*, OR (95%CI) and covariance matrix with SPSS21.0. Then input the B value and covariance matrix into the programmed excel. Figure out three indexes including Relative Excess Risk of Interaction (RERI), Attributable Proportion of interaction (AP), and Synergy index S) to evaluate gene-gene additive interactions. Where RERI and AP did not contain ‘0’ and S did not contain ‘1’ could be considered significant (Jabbarpoor Bonyadi et al., 2020). All hypothesis testing in this study was two-side, and *p* < 0.05 was statistically significant.

## Results

### Participants characteristics

Three groups of case-control studies were used in this study: NeC (840 cases) vs. HLD (1792 cases), SC (496 cases) vs. HLD (1792 cases) and CHB + LC (1,371 cases) vs. HCC (421 cases). The difference of the four factors including age, gender, the history of smoking and drinking in the three study groups were statistically significant (*p* < 0.001). Details are shown in Supplementary Table S3. The results of Hardy-Weinberg equilibrium test are shown in Supplementary Table S4. All 11 loci accorded with H-W equilibrium law (*p* > 0.05), which proved that the research sample was representative of the population. Before performing the correlation analysis, we calculated the genotypes frequency and the minor allele frequency of 11 SNPs in five groups (Supplementary Table S5).

### The results in NeC vs. HLD

The univariate analysis showed that *MSH5*-rs1150793 was related to HBV susceptibility. *MSH5*-rs1150793G) as a risk base (nominal *p* = 0.002, OR = 1.346) for HBV susceptibility, the co-dominant model and the recessive model had the same result that GG was a risk genotype (nominal *p* = 0.006, OR = 5.341; nominal *p* = 0.007, OR = 5.113, Supplementary Table S6 **)**. The results of multivariate Logistic Regression analysis showed that individuals carrying *MSH5*-rs1150793 (GG) or *MSH5*-rs1150793 (AG + GG) were more likely to be infected with HBV than AA in the co-dominant and the dominant model (nominal *p* < 0.05, OR >1). Details are shown in Table 1.

**TABLE 1:** The multivariate Logistic Regression analysis of predictive factors for HBV susceptibility under three models in NeC vs. HLD group.

Variable	B	S.E	Wald	*P*_*n*_	OR (95%CI)
Codominant					
Age	0.162	0.064	6.510	0.011*	1.176 (1.038,1.332)
sex (Male)	0.400	0.097	16.859	0.000*	1.492 (1.233,1.806)
smoke (Yes)	0.851	0.107	63.055	0.000*	2.341 (1.898,2.889)
*MSH5*-rs1150793			10.167	0.006*	
*MSH5*-rs1150793 (AG)	0.200	0.111	3.222	0.072	1.221 (0.982,1.520)
*MSH5*-rs1150793 (GG)	1.656	0.613	7.305	0.007*	5.237 (1.576,17.399)
*PMS1*-rs5742933			7.507	0.023*	
*PMS1*-rs5742933 (GC)	−0.156	0.096	2.676	0.102	0.855 (0.709,1.031)
*PMS1*-rs5742933(CC)	−0.488	0.199	6.006	0.014*	0.614 (0.416,0.907)
Dominant					
Age	0.161	0.063	6.450	0.011*	1.175 (1.037,1.330)
sex (Male)	0.402	0.097	17.13	0.000*	1.495 (1.236,1.809)
smoke (Yes)	0.847	0.107	62.824	0.000*	2.334 (1.893,2.878)
*MSH5*-rs1150793 (AG + GG)	0.257	0.110	5.498	0.019*	1.293 (1.043,1.603)
*PMS1*-rs5742933 (GC + CC)	−0.206	0.091	5.110	0.024*	0.814 (0.681,0.973)
Recessive					
Age	0.168	0.064	7.025	0.008*	1.183 (1.045,1.340)
sex (Male)	0.395	0.097	16.520	0.000*	1.485 (1.227,1.797)
smoke (Yes)	0.847	0.107	62.703	0.000*	2.333 (1.892,2.877)
*MSH5*-rs1150793 (GG)	1.632	0.612	7.113	0.008*	5.113 (1.541,16.960)
*PMS1*-rs5742933(CC)	−0.426	0.196	4.725	0.030*	0.653 (0.445,0.959)

Abbreviations: NeC, negative control group; HLD, HBV-induced liver diseases (LC + CHB + HCC). *P*_*n*_: nominal *p*-values. *: *P*_*n*_ < 0.05.

**FIGURE 1:**
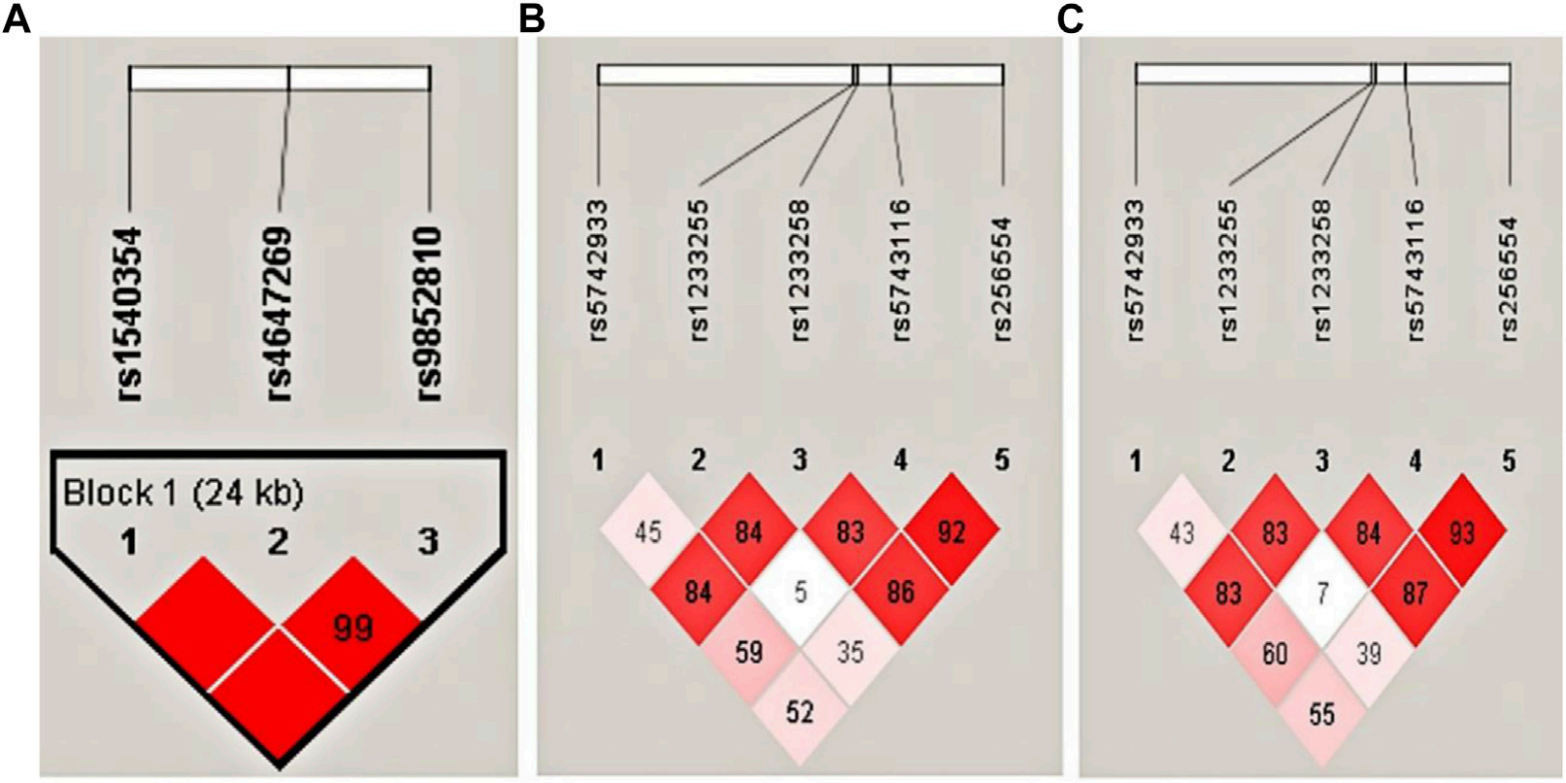
Haplotype analysis for *MLH1* and *PMS1* in NeC vs. HLD, SC vs. HLD and CHB + LC vs. HCC groups by Haploview4.2. **(A)** Three SNPs of *MLH1* in NeC vs. HLD, SC vs. HLD and CHB + LC vs. HCC groups; **(B)**. Five SNPs of *PMS1* in NeC vs. HLD and SC vs. HLD groups; **(C)**. Five SNPs of *PMS1* in CHB + LC vs. HCC group. This Figure was generated using our sample population. The color ranges from white to red, representing the linkage degree from low to high. The values in boxes are D' values, the larger the value is, the higher the degree of linkage between loci is.

Haplotype analysis was carried out on three loci of *MLH1* gene on chromosome three and five loci of *PMS1* gene on chromosome 2, respectively. Haplotype analysis showed that three SNPs near the *MLH1* gene formed one haploid domain composed of rs1540354, rs4647269 and rs9852810 on chromosome 3 (Figure 1), but these haplotypes had nothing to do with HBV infection (Table 2). No linkage disequilibrium was found in *PMS1* gene, shown in Figure 1. GMDR analysis did not find the optimal interaction model (Table 3). The results of gene-gene multiplicative interaction showed the positive interaction between *MLH1*-rs1540354 (AA + AT) and *PMS1*-rs1233255 (AA) to HBV susceptibility (nominal *p* = 0.024, OR = 1.240, Table 4). The results (Table 5) of gene-gene additive interaction analysis showed that *PMS1*-rs1233255 (AA) and *PMS1*-rs256554(CA + CC) were both promoting factors of HBV infection when acting alone. Co-exposure of the two loci was also related to HBV infection, and the effect value was not stronger than that of single exposure. The relative excess risk of interaction (RERI) of the two loci was −2.900 (-5.612, −0.188). When the two factors exist simultaneously, the risk of HBV infection was 0.283 times as high as the sum of their respective risk (S:0.283 (0.166, 0.483)). The other negative results of additive interaction analysis were seen in Supplementary Table S7.

**TABLE 2:** Haplotype analysis for three SNPs within *MLH1* gene in NeC vs. HLD, SC vs. HLD and CHB + LC vs. HCC by Haploview4.2.

Group	Haplotype	Freq	Case, Control Ratios	*p*-value	OR (95%CI)
NeC vs. HLD					
	Block 1				
	AGG	0.628	2227.8:1,334.2,1062.3:617.7	0.629	0.972 (0.862,1.096)
	TCG	0.305	1,091.2:2470.8,509.7:1,170.3	0.827	1.013 (0.893,1.149)
	ATA	0.067	243.0:3,319.0.108.0:1,572.0	0.595	1.066 (0.843,1.347)
SC vs. HLD					
	Block 1				
	ACG	0.634	2227.9:1,334.1,658.0:334.0	0.029*	0.848 (0.731,0.983)
	TCG	0.299	1,091.1:2470.9,270.0:722.0	0.038*	1.181 (1.009,1.381)
	ATA	0.067	243.0:3,319.0,64.0:928.0	0.681	1.062 (0.798,1.411)
CHB + LC vs. HCC					
	Block 1				
	ACG	0.625	538.0:300.0,1689.7:1,034.3	0.256	1.097 (0.934,1.289)
	TCG	0.306	241.0:597.0.850.3:1873.7	0.177	0.890 (0.751,1.055)
	ATA	0.068	59.0:779.0.184.0:2540.0	0.774	1.046 (0.771,1.417)

Abbreviations: Three SNPs, including rs1540354_T, rs4647269_T and rs9852810_A were in almost absolute LD. *: *p* < 0.05.

**TABLE 3:** Generalized multifactor dimension reduction analysis of 11 loci in NeC vs. HLD, SC vs. HLD and CHB + LC vs. HCC groups by GMDR (v0.9).

Group	Model	TBA	*P*	CVC
NeC vs. HLD				
	rs5742933	0.4926	0.6230	5/10
	rs1540354 rs1150793	0.4783	0.9453	2/10
	rs1150793 rs5742933 rs1233258	0.5042	0.3770	6/10
	rs1540354 rs5743933 rs256554 rs12112229	0.5234	0.1719	7/10
	rs1540354 rs175080 rs5742933 rs256554 rs1233258	0.5246	0.0547	5/10
SC vs. HLD				
	rs256554	0.5323	0.0107*	10/10
	rs175080 rs256554	0.5366	0.1719	9/10
	rs1540354 rs175080 rs256554	0.5280	0.1719	8/10
	rs1540354 rs175080 rs256554 rs1233258	0.5435	0.0547	7/10
	rs1540354 rs175080 rs256554 rs1233258 rs12112229	0.5756	0.0010*	10/10
CHB + LC vs. HCC				
	rs1540354	0.4791	0.9453	5/10
	rs175080rs256554	0.5005	0.3770	7/10
	rs1540354rs175080rs256554	0.4606	0.9453	4/10
	rs1540354rs4647269rs175080rs256554	0.4484	0.9893	2/10
	rs1540354rs4647269rs9852810rs256554rs12112229	0.4917	0.8281	3/10

Abbreviations: TBA, testing balanced accuracy; CVC, cross-validation consistency; The limit dimension was set to five; Confounding factors such as gender, age and the history of drinking and smoking were controlled in the operation. *: *p <* 0.05.

**TABLE 4:** The multiplicative interaction analysis of predictive factors in NeC vs. HLD, SC vs. HLD and CHB + LC vs. HCC groups by Logistic Regression model.

Group	Variable	B	S.E	Wald	*P*_*n*_	OR (95%CI)
NeC vs. HLD						
	age	0.178	0.063	7.896	0.005*	1.195 (1.055,1.354)
	sex (Male)	0.403	0.097	17.172	0.000*	1.496 (1.237,1.811)
	smoke (Yes)	0.838	0.107	61.697	0.000*	2.311 (1.875,2.849)
	rs1233255*rs1540354	0.215	0.095	5.113	0.024*	1.240 (1.029,1.493)
	rs1150793*rs5742933	0.358	0.137	6.884	0.009*	1.431 (1.095,1.870)
	rs1233258*rs5742933	0.208	0.104	4.025	0.045*	1.231 (1.005,1.508)
SC vs. HLD						
	drink (Yes)	0.945	0.116	65.860	0.000*	2.572 (2.048,3.232)
	rs1540354*rs5743116	−0.998	0.476	4.405	0.036*	0.368 (0.145,0.936)
	rs12112229*rs1540354	0.230	0.116	3.947	0.047*	1.258 (1.003,1.578)
	rs1233258*rs256554	0.429	0.130	10.867	0.001*	2.572 (2.048,3.232)
CHB + LC vs. HCC						
	age	0.992	0.101	97.193	0.000*	2.697 (2.214,3.285)
	sex (Male)	0.331	0.156	4.510	0.034*	1.393 (1.026,1.890)
	smoke (Yes)	0.618	0.134	21.318	0.000*	1.856 (1.427,2.413)
	rs1233258*rs5742933	0.526	0.269	3.835	0.050	1.693 (1.000,2.867)

Genotype assignment based on the optimum risk association results of genetic models.

NeC vs. HLD: rs5742933: GG 1), GC + CC 0); rs1540354: AA + AT 1), TT 0); rs1150793: AA 0), GG + AG 1); rs1233255: AA 1), AC + CC 0); rs1233258: CC + CT 1), TT 0).

SC, vs. HLD: rs1540354: AA + AT 0), TT 1); rs12112229: CC + CA 1), AA 0); rs5743116: TT 0), TC + CC 1); rs256554: CC 0), CA + AA 1); rs1233258: TT + CT 1), CC 0).

CHB + LC, vs. HCC: rs5742933: GG + GC 0), CC 1); rs1233258: TT + CT 1), CC 0).

*P*_*n*_: nominal *p*-values. *: *P*_*n*_ < 0.05.

**TABLE 5:** The positive results of additive interaction analysis of 11 loci and four other factors in NeC vs. HLD group.

SNP1	SNP2	HLD	NeC	B	*P*	OR (95%CI)	RERI/AP/S
rs5742933	rs5743116						
GC + CC	TC + CC	318	249			1.0	
GG	TT	331	101	1.136	0.000	3.115 (2.273,4.267)	RERI: 3.839(-5.167,-2.512)
GC + CC	TC + CC	344	87	1.181	0.000	3.258 (2.371,4.477)	AP: 2.505(-3.322,-1.688)
GG	TT	747	392	0.427	0.001	1.533 (1.181,1.991)	S:0.122(0.075,0.199)
rs1233255	rs256554						
AC + CC	AA	30	34			1.0	
AA	CA + CC	394	203	0.709	0.011	2.033 (1.174,3.521)	RERI: 2.900(-5.612,-0.188)
AC + CC	AA	74	23	1.390	0.000	4.015 (1.936,8.326)	AP: 1.351(-2.291,-0.410)
AA	CA + CC	1,209	569	0.764	0.005	2.148 (1.257,3.670)	S:0.283(0.166,0.483)
rs5743116	rs1233258						
TC + CC	TT	203	21			1.0	
TT	TC + CC	464	317	−1.692	0.000	0.184 (0.112,0.303)	RERI:1.025(0.956,1.095)
TC + CC	TT	462	281	−1.744	0.000	0.175 (0.107,0.285)	AP:2.667(1.463,3.872)
TT	TC + CC	617	214	−0.956	0.000	0.384 (0.233,0.634)	S:0.375(0.305,0.462)

Abbreviations: HLD, HBV-induced liver disease (CHB + LC + HCC); RERI, Relative Excess Risk of Interaction; AP, Attributable Proportion of interaction; S: Synergy index. When calculating covariance matrix, take SNPs, other than the analysis SNPs, together with gender, age, the history of drinking and smoking as control variables. The bold font shows statistical significance.

### The results in SC vs. HLD

The univariate analysis showed that *MLH1*-rs1540354 was related to the spontaneous clearance of HBV. *MLH1*-rs1540354T) as a risk base (nominal *p* = 0.039, OR = 1.180), individuals with TT were more difficult to spontaneously clear HBV in the co-dominant and the recessive models (nominal *p* = 0.005, OR = 1.925; nominal *p* = 0.005, OR = 1.896). Details are shown in Supplementary Table S6. In the results of multivariate analysis, *MLH1*-rs1540354 (TT) was also a risk factor for the spontaneous clearance of HBV in the co-dominant and recessive model (nominal *p* < 0.05, OR > 1, Table 6).

**TABLE 6:** The multivariate Logistic Regression analysis of predictive factors for the ability of HBV spontaneous clearance under three models in SC vs. HLD group.

Variable	B	S.E	Wald	*P*_*n*_	OR (95%CI)
Codominant					
drink (Yes)	0.954	0.116	67.349	0.000*	2.596 (2.067,3.261)
*MLH1*-rs1540354			8.040	0.018*	
*MLH1*-rs1540354 (AT)	−0.059	0.112	0.284	0.594	0.942 (0.757,1.172)
*MLH1*-rs1540354 (TT)	0.634	0.244	6.766	0.009*	1.885 (1.169,3.040)
*PMS2*-rs12112229			7.604	0.022*	
*PMS2*-rs12112229 (CA)	−0.193	0.128	2.290	0.130	0.824 (0.642,1.059)
*PMS2*-rs12112229 (AA)	−1.023	0.425	5.808	0.016*	0.359 (0.156,0.826)
Dominant					
drink (Yes)	0.938	0.116	65.455	0.000*	2.556 (2.036,3.208)
*PMS1*-rs256554 (CA + AA)	0.721	0.224	10.328	0.001*	2.056 (1.325,3.191)
*PMS1*-rs5743116 (TC + CC)	−0.594	0.227	6.865	0.009*	0.552 (0.354,0.861)
Recessive					
drink (Yes)	0.945	0.116	66.441	0.000*	2.573 (2.050,3.229)
*MLH1*-rs1540354 (TT)	0.649	0.237	7.459	0.006*	1.913 (1.201,3.046)
*PMS2*-rs12112229 (AA)	−0.970	0.423	5.248	0.022*	0.379 (0.165,0.869)

Abbreviations: SC, spontaneous clearance; HLD, HBV-induced liver diseases (LC + CHB + HCC). *P*_*n*_: nominal *p*-values. *: *P*_*n*_ < 0.05.

As shown as Table 2, three SNPs near the *MLH1* gene form a haploid domain composed of rs1540354_T, rs4647269_T and rs9852810_A. The frequency of risk haplotype TCG (rs1540354T, rs4647269C, rs9852810G) in HLD group was higher than SC group (*p* = 0.038, OR = 1.181).

In GMDR analysis, rs1540354 rs175080 rs256554 rs1233258 rs12112229 constituted the optimal interaction model related to the spontaneous clearance of HBV (*p* = 0.001, Table 3). According to the 5-factor interaction combination, the participants were re-divided into ‘high risk’ and ‘low risk’ groups. 68 combinations were defined as ‘high risk’ and 41 combinations as ‘low risk’, as shown in the Supplementary Figure S1. The ability of spontaneous clearance of HBV in ‘low risk’ group was 2.905 times that of ‘high risk’ group. Table 4 showed the results of gene-gene multiplicative interaction by Logistic Regression analysis. There was positive multiplicative interaction between *PMS2*-rs12112229(CC + CA) and *MLH1*-rs1540354 (TT) (nominal *p* = 0.047, OR = 1.258). No statistically significant result was found in the additive interaction analysis (Supplementary Table S8).

### The results in CHB + LC vs. HCC

Univariate analysis showed that *PMS2*-rs12112229 was associated with cancerization. In the co-dominant model, individuals with CA were more likely to suffer from HBV-HCC after HBV infection than those with CC (nominal *p* = 0.042, OR = 1.304, Supplementary Table S6). Multivariate analysis did not find any locus associated with cancerization after HBV infection (Supplementary Table S9). In addition, haplotype analysis and three kinds of interaction analysis also did not find genetic factors related to cancerization. See Tables 2–4; Supplementary Table S10.

## Discussion

This study investigated the relationship between the occurrence and development of HBV infection and 11 loci in five genes. The analysis of baseline showed that age, gender (male) and the history of smoking were risk factors for HBV infection and deterioration. This result was consistent with the results of other research teams. Studies have shown that HBV susceptibility factors include gender (male) (Si et al., 2019) and advanced age (Desalegn et al., 2019). In addition, smoking may increase susceptibility to HBV-HCC by affecting viral load (Wang et al., 2019).

The results in NeC vs. HLD group showed that *MSH5*-rs1150793G) as a risk base, GG and AG + GG were risk genotypes for HBV infection. According to the database eQTLGen Consortium (eQTLGen - cis-eQTLs), *MSH5*-rs1150793 (chr6: 31717696) was related to the expression of *HLA-C* gene (chr6: 31238216). Meder also found a highly significant association between their lead-SNP rs9262636 and *HLA-C* mRNA levels (*p* = 4.05 × 10^–47^) (Meder et al., 2014). For *HLA-C*, mRNA levels decreased with each additional G allele of rs9262636. Thus we speculated that the mutant base G of rs1150793 could affect the normal expression of *HLA-C*. HLA-C belongs to heavy chain homologue of HLA-Ⅰ. HLA-Ⅰ molecules are important participants in cytotoxic T cell reaction. Specifically, at the immune level, HLA-Ⅰ molecules present the peptides from cytoplasmic proteolysis, and it is recognized by CD8+T cells to initiate adaptive immune response. Although expressed on the cell surface about ten times lower than HLA-A and B, HLA-C represents a potentially particular target for the mechanisms put in place by viral infections, acting as a ligand for both T cell receptors and NK cell receptors (Apps et al., 2016; Vollmers et al., 2022). Moreover, HLA-Cw*03-restricted CD8+T cells have been shown to induce escape mutations in HIV as a result of immune pressure, demonstrating that HLA-C-restricted T cells are involved in defence against viral infections (Honeyborne et al., 2010; Leslie et al., 2010). Because of the low expression of HLA-C, the antigen presenting cells and T cells cannot play their roles in time, which leads to immune escape of HBV.

In NeC vs. HLD group, a multiplication interaction between *MLH1*-rs1540354 (AA + AT) and *PMS1*-rs1233255 (AA) to HBV susceptibility was calculated, and it was a positive interaction. In biology, it showed the synergistic effect of the two. In the results of gene-gene additive interaction analysis, effect of interaction between the risk genotype AA of *PMS1*-rs1233255 and the risk genotype CA + CC of *PMS1*-rs256554 was lower than the sum of individual effects. In biology, it showed the antagonism between them.

**FIGURE 2:**
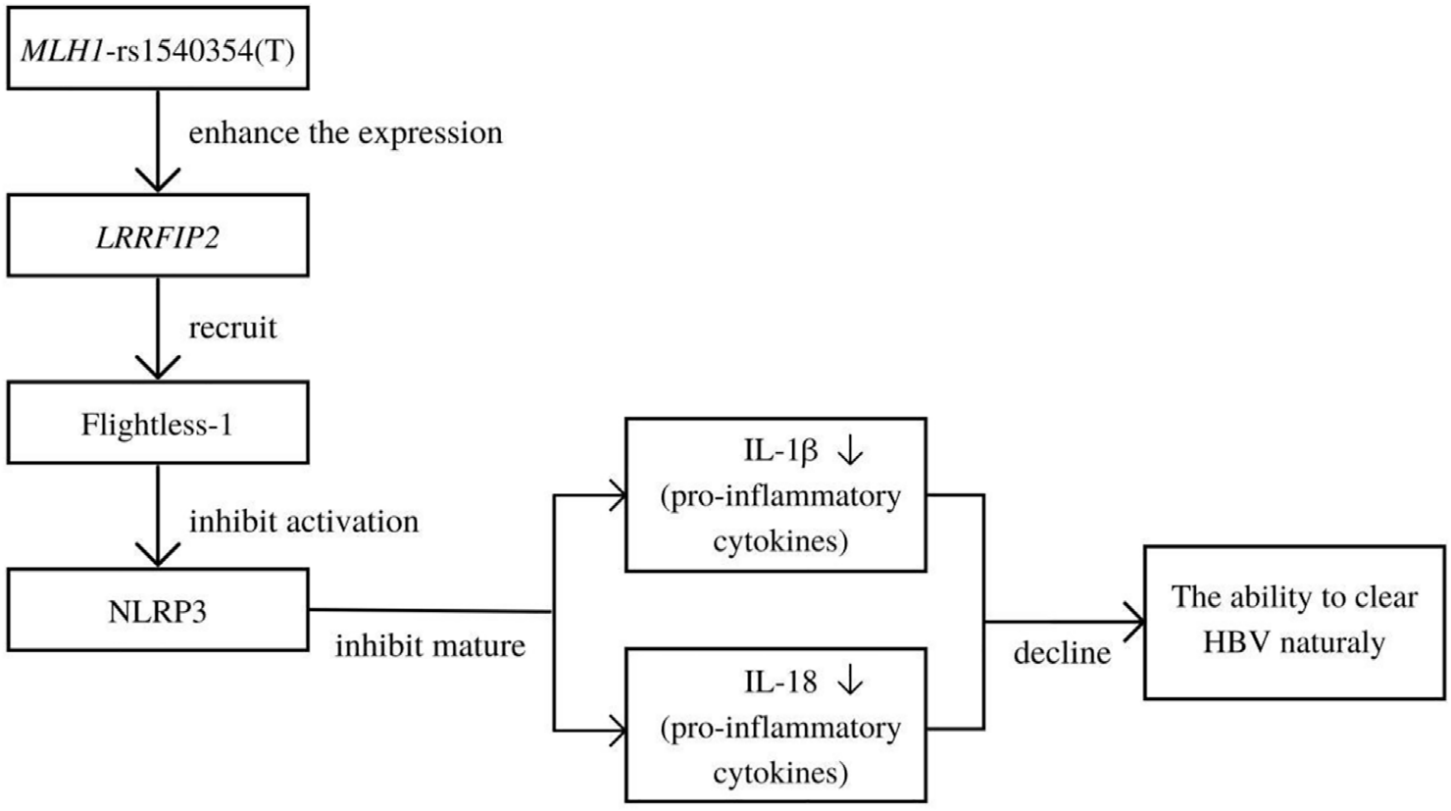
Mechanism of *MLH1*-rs1540354(T) mutant affecting human ability to spontaneously clear HBV.

In the study of factors affecting spontaneous clearance of HBV, univariate, multivariate and interaction analyses results all showed that *MLH1*-rs1540354T) was a risk factor. Further analysis based on the eQTLGen Consortium showed that the expression level of *LRRFIP2 (leucine-rich repeat FliⅠ-interaction protein* 2) was related to the polymorphism of *MLH1*-rs1540354. As shown as Figure 2, the protein transcribed and translated by *LRRFIP2* is related to NLRP3 (NLR family pyrin domain containing 3). NLRP3 is the most characteristic inflammasome activated by the infection or stress reaction of cells, which is responsible for the maturation of pro-inflammatory cytokines IL-1β and IL-18 (Jin et al., 2013). IL-1β and IL-18 are proven pro-inflammatory cytokines, which can participate in innate immune response and inhibit HBV infection (Motavaf et al., 2014; Wei et al., 2021). Previously, Stefan J Schunk confirmed through eQTLGen Consortium and a series of experiments that *NLRP3*-rs10754555G can enhance the expression of *NLRP3* mRNA and further make the body release more IL-1β (Schunk et al., 2021). Moreover, other studies have shown that LRRFIP2 can inhibit NLRP3 activation by recruiting Flightless one into the NLRP3 inflammasome (Burger et al., 2016). Therefore, we made a hypothesis that the risk base T of *MLH1*-rs1540354 might promote HBV infection and HCC by enhancing the expression of *LRRFIP2* and inhibiting the anti-inflammatory function of NLRP3.

Take CHB + LC group as control group and HCC group as case group to study the relationship between cancerization and loci. The multivariate analysis showed *MLH1*-rs1540354 was not associated with cancerization, which was in accordance with Zhu’s study result that *MLH1*-rs1540354 had nothing to do with HCC (Zhu et al., 2017).

*PMS2*-rs12112229A) was a risk base for the deterioration of HBV infection, especially cancerization. *PMS2*-rs12112229 is located at the intron variant of chr7. By searching the database eQTLGen Consortium, *PMS2*-rs12112229 (chr7: 6036515) was related to the expression of small nucleolar RNA (*SNORA42*, chr7:6056575). Wang G proved that SNORA42 can promote HCC cells proliferation and inhibit their apoptosis through the experiment of the *SNORA42* knockdown in hepatoma cells (Wang et al., 2021). And this mechanism is achieved through the P53 signaling pathway and the cell cycle. P21, a cyclin-dependent kinases inhibitor (CDKI), locates at the downstream of p53 and is activated in response to DNA damage (Shamloo and Usluer, 2019). The p53-p21 axis is a significant tumor suppressor, and it is usually disrupted during cancer progression. Through the study of database eQTLGen Consortium and the related mechanisms, we predict mutant base A can upregulate *SNORA42* expression.

Association methods based on linkage disequilibrium (LD) offer a promising approach for detecting genetic variations that are responsible for complex human diseases (Liu et al., 2008). In this study, we discovered that the risk haplotype TCG (rs1540354T, rs4647269C, rs9852810G) was more closely associated with chronic HBV infection. Through the database Ensembl, we found that there was linkage disequilibrium between *MLH1*-rs1540354 and *MLH1*-rs4647269 in other population, such as the British and Italian, and similar situations existed in *MLH1*-1540,354 and *MLH1*-9852,810. Although the association between this haplotype and disease has not been found, these data preliminarily confirm the credibility of our research results.

There were some advantages in our research, first, most of our loci were the first time to study the relationship with HBV occurrence and development, which was innovative. Then, in addition to single-locus univariate and multivariate analysis, we also conducted haplotype analysis and three kinds of interaction analyses. Explore the relationship between candidate SNPs and the occurrence and development of HBV infection from more dimensions and aspects by various methods. Thirdly, the sample size of this study was large enough, which could effectively reduce the possibility of making class II mistakes and increase the power of test. However, this research still had some limitations. First of all, all the included samples were from Hebei Province, China. And as this study used multivariable regression models, such models may have issues with multiple testing, results should be interpreted with caution. Further researches with larger sample sizes in multiple centers were necessary to confirm these findings. Secondly, this study was more based on statistical analysis. So, we will carry out research on molecular biology to prove the reliability of the results in the following studies.

## Data Availability

The data presented in the study are deposited in the Figshare repository (https://figshare.com/). Accession Number 10.6084/m9.figshare.21525174.
