# Supplementary material for: Comprehensive investigating of mismatch repair genes (MMR) polymorphisms in participants with chronic hepatitis B virus infection
Source: Front Genet. 2023 Feb 1;14:1077297. doi: 10.3389/fgene.2023.1077297 (PMC9928949; doi:10.3389/fgene.2023.1077297)
Supplement: Supplementary file 1 [file Table1.pdf]

Supplementary Material

1 Supplementary Figures and Tables

1.1 Supplementary Tables

Supplementary table1: The basic information of selected SNPs in Han Chinese People from Ensembl.

Gene	SNP	Location in Gene Region	Function Prediction	MAF	Alleles
MLH1	rs1540354	intron variant	*eQTL/Risk-SNP*	0.325	A>T
MLH1	rs4647269	intron variant	*eQTL/Risk-SNP/enhancer*	0.050	C>T
MLH1	rs9852810	intron variant	*eQTL/Risk-SNP/enhancer*	0.050	G>A
MLH3	rs175080	missense variant	*eQTL/nsSNP/Risk-SNP/enhancer*	0.160	G>A
MSH5	rs1150793	intron variant	*eQTL/TFBS/Risk-SNP/super-enhancer/enhancer*	0.102	A>G
PMS1	rs5742933	5 prime UTR variant	*eQTL/*TFBS/Splicing(ESE or ESS) */Risk-SNP/super-*	0.204	G>C
PMS1	rs256554	intron variant	*eQTL/Risk-SNP/enhancer*	0.214	C>A
PMS1	rs1233255	intron variant	*eQTL/TFBS/Risk-SNP/super-enhancer/enhancer*	0.107	A>C
PMS1	rs1233258	intron variant	*eQTL/TFBS/Risk-SNP/super-enhancer/enhancer*	0.364	T>C
PMS1	rs5743116	intron variant	*eQTL/TFBS/Risk-SNP/super-enhancer/enhancer*	0.194	T>C
PMS2	rs12112229	intron variant	*eQTL/TFBS/Risk-SNP/super-enhancer/enhancer*	0.083	C>A

Abbreviations: MAF, minor allele frequency; UTR, untranslated region; ESE, Exonic Splicing Enhancer; ESS, Exonic Splicing Silencer; TFBS, transcription factor binding sites; SNPs, single nucleotide polymorphisms.

Supplementary table2: Primer sequences of 11 SNPs for MassARRAY allelic discrimination.

Polymorphism		Sequence
rs1540354	F-PCRP	ACGTTGGATGTCGCCTAGCTGTAGGTTATC
	R-PCRP	ACGTTGGATGCTGGTACACAGAAATCCCTC
	UEP_SEQ	tgCACAGAAATCCCTCAATAAATCT
rs4647269	F-PCRP	ACGTTGGATGAGTCACAACCACTTTTCCTG
	R-PCRP	ACGTTGGATGTCTGTTGTCCTGTGTTGCTG
	UEP_SEQ	aacgGTGTTGCTGTAGACAAGGATAC
rs9852810	F-PCRP	ACGTTGGATGTATGGAGCATCTACGGTGTG
	R-PCRP	ACGTTGGATGACTTTCCCTGCAGGGATAAG
	UEP_SEQ	GGATAAGAGCATTAAATGAGATAA
rs175080	F-PCRP	ACGTTGGATGGCAAGCCACATCCTTAACTC
	R-PCRP	ACGTTGGATGGGGTCATAGGACTTTCTCTC
	UEP_SEQ	ccccgCATAGGACTTTCTCTCAAACTA
rs1150793	F-PCRP	ACGTTGGATGAATCCTTCCCCTACCTCACC
	R-PCRP	ACGTTGGATGCTGATTACCTGGAGATGACC
	UEP_SEQ	cTCCTAAATTAACTCCTACAAGA
rs5742933	F-PCRP	ACGTTGGATGCGGCTAGTGGATGGTAATTG
	R-PCRP	ACGTTGGATGATACCCGTTAGTCACACCAC
	UEP_SEQ	gtatTCACACCACACTACCTT
rs256554	F-PCRP	ACGTTGGATGCATAAATAATCCCCTCAGAAG
	R-PCRP	ACGTTGGATGGATGAGATCAGGACTTAGAA
	UEP_SEQ	gccAGTATGTAATATAAAGGTAGCATTA
rs1233255	F-PCRP	ACGTTGGATGGGAATAGGTAAAAGATCTTC
	R-PCRP	ACGTTGGATGCTGCTTGGGAAAGCAGAGAT
	UEP_SEQ	AAGACAAATAACTCAACATTTAGT
rs1233258	F-PCRP	ACGTTGGATGAGGGCTTGGGAGAAAACATC
	R-PCRP	ACGTTGGATGTCTGTGACCACTCCTTATAG
	UEP_SEQ	ccccGTGACCACTCCTTATAGGTTCCT
rs5743116	F-PCRP	ACGTTGGATGCAAAGAGAAGACAGATGTCA
	R-PCRP	ACGTTGGATGTTGTGAGGCATTTCCCATCG
	UEP_SEQ	ATGGAGCTCCTGAAGTTA

rs12112229	F-PCRP	ACGTTGGATGGCAACTGACTCCTTTGTATA
	R-PCRP	ACGTTGGATGCGCCTGGCTAGGGATAATAG
	UEP_SEQ	TGTGAGCAATGCTGT

Abbreviations: F-PCRP, Forward primers; R-PCRP, Reverse primers; UEP_SEQ, UEP primers

Supplementary table3: The baseline characteristics of the participants in five groups.

Factors	NeC N=840	SC N=496	HCC N=421	CHB+LC N=1371	HLD N=1792	NeC vs. HLD		SC vs. HLD		CHB+LC vs. HCC	
	n (%)	n (%)	n (%)	n (%)	n (%)	$\chi 2$	P_1	$\chi 2$	P_2	$\chi 2$	P_3
Age						55.376	< 0.001*	25.250	< 0.001*	144.950[a]	< 0.001*
1-20	2(0.2)	8(1.6)	0	3(0.2)	3(0.2)						
21-40	268(31.9)	122(24.6)	13(3.1)	343(25.0)	356(19.9)						
41-60	358(42.6)	241(48.6)	253(60.1)	751(54.8)	1004(56.0)						
61-100	212(25.2)	125(25.2)	155(36.8)	274(20.0)	429(23.9)						
Gender						62.281	< 0.001*	22.729	< 0.001*	15.717	< 0.001*
Male	454(54.0)	290(58.5)	327(77.7)	926(67.5)	1251(69.8)						
Female	386(46.0)	206(41.5)	94(22.3)	445(32.5)	541(30.2)						
Smoke						122.449	< 0.001*	44.427	< 0.001*	50.942	< 0.001*
Y	179(21.3)	134(27.0)	247(58.7)	534(38.9)	781(43.6)						
N	661(78.7)	362(73.0)	174(41.3)	837(61.1)	1011(56.4)						
Drink						67.157	< 0.001*	60.374	< 0.001*	32.795	< 0.001*
Y	281(33.5)	153(30.8)	264(62.7)	641(46.8)	905(50.5)						
N	559(66.5)	343(69.2)	157(37.3)	730(53.2)	887(49.5)						

Abbreviations: NeC, negative control; SC, spontaneous clearance; CHB, chronic hepatitis B; LC, liver cirrhosis; HCC, hepatocellular carcinoma; HLD, HBV-induced liver diseases (CHB+LC+HCC). a: Fisher exact probability method. *: $P < 0.05$.

Supplementary table4: The test of Hardy-Weinberg Equilibrium for the 11 SNPs in all individuals.

SNP	Genotype	Number	MAF	χ^2	*P*
MLH1-rs1540354	AA	1511	0.300	1.012	0.314
	AT	1329			
	TT	268			
MLH1-rs4647269	CC	2710	0.067	0.338	0.561
	TC	385			
	TT	16			
MLH1-rs9852810	GG	2720	0.066	0.451	0.502
	GA	381			
	AA	16			
MLH3-rs175080	GG	2037	0.180	0.324	0.569
	GA	885			
	AA	103			
MSH5-rs1150793	AA	2408	0.120	0.119	0.730
	AG	663			
	GG	43			
PMS1-rs5742933	GG	1889	0.220	0.183	0.669
	GC	1055			
	CC	154			
PMS1-rs256554	CC	1776	0.241	0.511	0.475
	CA	1107			
	AA	185			
PMS1-rs1233255	AA	2292	0.139	0.118	0.732
	AC	735			
	CC	62			
PMS1-rs1233258	TT	1159	0.389	0.028	0.868
	TC	1470			
	CC	472			
PMS1-rs5743116	TT	1902	0.213	1.873	0.171
	TC	1060			

	CC	127			
PMS2-rs12112229	CC	2387	0.122	2.977	0.084
	CA	687			
	AA	36			

Abbreviations: MAF, minor allele frequency; SNPs, single nucleotide polymorphisms.

Supplementary table5: The genotypes frequency and the minor allele frequency of 11 SNPs in five groups.

Genotype	NeC (N=840)	SC (N=496)	HCC (N=421)	CHB+LC (N=1371)	HLD (N=1792)
MLH1-rs1540354					
AA	417(49.6%)	250(50.4%)	212(50.4%)	632(46.6%)	844(47.6%)
AT	333(39.6%)	222(44.8%)	173(41.1%)	601(44.3%)	774(43.6%)
TT	88(10.5%)	24(4.8%)	33(7.8%)	123(9.1%)	156(8.8%)
A	1167	722	597	1865	2462
T	509	270	239	847	1086
MAF (T allele)	0.303699	0.272177	0.285885	0.312316	0.306088
MLH1-rs4647269					
CC	730(86.9%)	434(87.5%)	364(86.5%)	1182(86.7%)	1546(86.7%)
CT	94(11.2%)	60(12.1%)	54(12.8%)	177(13.0%)	231(12.9%)
TT	7(0.8%)	2(0.4%)	3(0.7%)	4(0.3%)	7(0.4%)
C	1554	928	782	2541	3323
T	108	64	60	185	245
MAF (T allele)	0.064982	0.064516	0.071259	0.067865	0.068666
MLH1-rs9852810					
GG	739(88.0%)	434(87.5%)	364(86.5%)	1183(86.9%)	1547(86.8%)
GA	94(11.2%)	58(11.7%)	54(12.8%)	175(12.8%)	229(12.8%)
AA	7(0.8%)	2(0.4%)	3(0.7%)	4(0.3%)	7(0.4%)

G	1572	926	782	2541	3323
A	108	62	60	183	243
MAF (A allele)	0.064286	0.062753	0.071259	0.067181	0.068144
MLH3-rs175080					
GG	542(64.5%)	316(63.7%)	271(64.4%)	908(67.6%)	1179(67.2%)
GA	237(28.2%)	130(26.2%)	124(29.5%)	394(29.3%)	518(29.5%)
AA	31(3.7%)	14(2.8%)	17(4.0%)	41(3.1%)	58(3.3%)
G	1321	762	666	2210	2876
A	299	158	158	476	634
MAF (A allele)	0.184568	0.171739	0.191748	0.177215	0.180627
MSH5-rs1150793					
AA	676(80.5%)	382(77.0%)	316(75.1%)	1034(76.1%)	1350(75.9%)
AG	161(19.2%)	106(21.4%)	96(22.8%)	300(22.1%)	396(22.3%)
GG	3(0.4%)	8(1.6%)	8(1.9%)	24(1.8%)	32(1.8%)
A	1513	870	728	2368	3096
G	167	122	112	348	460
MAF (G allele)	0.099405	0.122984	0.133333	0.12813	0.129359
PMS1-rs5742933					
GG	479(57.0%)	298(60.1%)	262(62.2%)	850(62.7%)	1112(62.7%)
GC	301(35.8%)	170(34.3%)	129(30.6%)	455(33.6%)	584(32.9%)
CC	52(6.2%)	24(4.8%)	27(6.4%)	51(3.8%)	78(4.4%)
G	1259	766	653	2155	2808

C	405	218	183	557	740
MAF (C allele)	0.243389	0.221545	0.2189	0.205383	0.208568
PMS1-rs256554					
CC	469(55.8%)	316(63.7%)	227(53.9%)	764(57.2%)	991(56.9%)
CA	305(36.3%)	154(31.0%)	157(37.3%)	491(36.8%)	648(37.2%)
AA	57(6.8%)	24(4.8%)	23(5.5%)	81(6.1%)	104(6.0%)
C	1243	786	611	2019	2630
A	419	202	203	653	856
MAF (A allele)	0.252106	0.204453	0.249386	0.244386	0.245554
PMS1-rs1233255					
AA	599(71.3%)	372(75.0%)	307(72.9%)	1014(75.6%)	1321(75.3%)
AC	218(26.0%)	116(23.4%)	99(23.5%)	302(22.5%)	401(22.8%)
CC	21(2.5%)	8(1.6%)	7(1.7%)	26(1.9%)	33(1.9%)
A	1416	860	713	2330	3043
C	260	132	113	354	467
MAF (C allele)	0.155131	0.133065	0.136804	0.131893	0.133048
PMS1-rs1233258					
TT	302(36.0%)	180(36.3%)	154(36.6%)	523(38.4%)	667(37.7%)
TC	408(48.6%)	230(46.4%)	198(47.0%)	634(46.6%)	832(47.0%)
CC	123(14.6%)	78(15.7%)	67(15.9%)	204(15.0%)	271(15.3%)
T	1012	590	506	1680	2166
C	654	386	332	1042	1374

MAF (C allele)	0.392557	0.395492	0.396181	0.382807	0.388136
PMS1-rs5743116					
TT	495(58.9%)	320(64.5%)	255(60.6%)	832(62.0%)	1087(61.8%)
TC	308(36.7%)	162(32.7%)	135(32.1%)	455(33.9%)	590(33.6%)
CC	32(3.8%)	14(2.8%)	25(5.9%)	56(4.2%)	81(4.6%)
T	1298	802	645	2119	2764
C	372	190	185	567	752
MAF (C allele)	0.222754	0.191532	0.222892	0.211095	0.213879
PMS2-rs12112229					
CC	645(76.8%)	368(74.2%)	308(73.2%)	1066(78.3%)	1374(77.1%)
CA	178(21.2%)	118(23.8%)	107(25.4%)	284(20.9%)	391(22.0%)
AA	10(1.2%)	10(2.0%)	4(1.0%)	12(0.9%)	16(9.0%)
C	1468	854	723	2416	3139
A	198	138	115	308	423
MAF (A allele)	0.118848	0.139113	0.137232	0.113069	0.118754

Abbreviations: NeC, negative control; SC, spontaneous clearance; CHB, chronic hepatitis B; LC, liver cirrhosis; HCC, hepatocellular carcinoma; HLD, HBV-induced liver diseases (CHB+LC+HCC); MAF, minor allele frequency.

Supplementary table6: The univariate analysis of predictive factors with regard to the risk of HBV infection, spontaneous clearance and cancerization between three independent sample case-control studies.

Loci	NeC vs. HLD		SC vs. HLD		CHB+LC vs. HCC	
	P_n	OR (95%CI)	P_n	OR (95%CI)	P_n	OR (95%CI)
MLH1-rs1540354						
AA		1		1		1
AT	0.119	1.148(0.965,1.367)	0.759	1.033(0.841,1.268)	0.192	0.858(0.682,1.080)
TT	0.364	0.876(0.658,1.166)	0.005*	1.925(1.225,3.027)	0.291	0.800(0.528,1.211)
AA/TA+TT	0.297	1.091(0.926,1.286)	0.265	1.120(0.918,1.367)	0.141	0.848(0.681,1.056)
AA+TA/TT	0.162	0.822(0.624,1.082)	0.005*	1.896(1.219,2.950)	0.458	0.859(0.575,1.283)
A/T	0.861	1.011(0.891,1.147)	0.039*	1.180(1.008,1.380)	0.147	1.134(0.957,1.345)
MLH1-rs4647269						
CC		1		1		1
CT	0.254	1.160(0.899,1.498)	0.616	1.081(0.798,1.464)	0.955	0.991(0.715,1.373)
TT	0.162	0.472(0.165,1.351)	0.983	0.983(0.203,4.747)	0.245	2.435(0.543,10.932)
CC/TC+TT	0.400	1.113(0.868,1.427)	0.624	1.078(0.799,1.453)	0.891	1.023(0.743,1.408)
CC+TC/TT	0.152	0.464(0.162,1.326)	0.973	0.973(0.201,4.698)	0.244	2.438(0.544,10.939)
C/T	0.621	1.061(0.839,1.341)	0.646	0.935(0.704,1.243)	0.734	0.949(0.702,1.283)
MLH1-rs9852810						
GG		1		1		1
GA	0.245	1.164(0.901,1.503)	0.514	1.108(0.815,1.506)	0.986	1.003(0.723,1.391)

AA	0.168	0.478(0.167,1.367)	0.982	0.982(0.203,4.744)	0.245	2.437(0.543,10.941)
GG/GA+AA	0.387	1.116(0.870,1.432)	0.524	1.103(0.815,1.494)	0.834	1.035(0.751,1.426)
GG+GA/AA	0.158	0.469(0.164,1.341)	0.969	0.970(0.201,4.682)	0.245	2.437(0.543,10.931)
G/A	0.602	1.064(0.842,1.346)	0.549	0.916(0.686,1.221)	0.681	0.939(0.694,1.270)
MLH3-rs175080						
GG		1		1		1
GA	0.960	1.005(0.836,1.208)	0.573	1.068(0.850,1.343)	0.669	1.054(0.827,1.345)
AA	0.509	0.860(0.550,1.346)	0.731	1.110(0.611,2.017)	0.268	1.389(0.777,2.485)
GG/AG+AA	0.894	0.988(0.828,1.179)	0.537	1.072(0.860,1.337)	0.488	1.086(0.860,1.372)
GG+AG/AA	0.502	0.859(0.551,1.339)	0.778	1.089(0.602,1.970)	0.288	1.367(0.768,2.432)
G/A	0.734	0.974(0.836,1.134)	0.531	1.063(0.878,1.288)	0.343	0.908(0.744,1.109)
MSH5-rs1150793						
AA		1		1		1
AG	0.047*	1.232(1.003,1.512)	0.654	1.057(0.829,1.347)	0.731	1.047(0.806,1.361)
GG	0.006*	5.341(1.630,17.505)	0.757	1.132(0.517,2.477)	0.834	1.091(0.485,2.452)
AA/AG+GG	0.010*	1.307(1.067,1.600)	0.615	1.062(0.839,1.345)	0.705	1.050(0.814,1.354)
AA+AG/GG	0.007*	5.113(1.561,16.746)	0.780	1.118(0.512,2.442)	0.853	1.079(0.481,2.421)
A/G	0.002*	1.346(1.116,1.624)	0.595	1.060(0.856,1.311)	0.695	1.047(0.833,1.316)
PMS1-rs5742933						
GG		1		1		1
GC	0.045*	0.836(0.701,0.996)	0.447	0.921(0.744,1.140)	0.494	0.920(0.724,1.169)
CC	0.020*	0.646(0.448,0.933)	0.569	0.871(0.542,1.401)	0.029*	1.718(1.056,2.794)

GG/GC+CC	0.013*	0.808(0.683,0.955)	0.392	0.914(0.745,1.122)	0.999	1.000(0.797,1.255)
GG+GC/CC	0.044*	0.690(0.481,0.990)	0.649	0.897(0.561,1.433)	0.020*	1.767(1.093,2.855)
G/C	0.005*	0.819(0.713,0.941)	0.378	0.926(0.781,1.099)	0.758	1.020(0.901,1.154)
PMS1-rs256554						
CC		1		1		1
CA	0.951	1.005(0.844,1.198)	0.008*	1.342(1.080,1.666)	0.537	1.076(0.853,1.358)
AA	0.399	0.863(0.614,1.215)	0.170	1.382(0.871.2.192)	0.855	0.956(0.588,1.554)
CC/CA+AA	0.841	0.983(0.832,1.161)	0.005*	1.347(1.096,1.656)	0.615	1.059(0.847,1.325)
CC+CA/AA	0.382	0.862(0.617,1.203)	0.350	1.243(0.788,1.960)	0.759	0.928(0.576,1.495)
C/A	0.611	0.966(0.844,1.105)	0.007*	1.266(1.065,1.505)	0.772	1.027(0.857,1.232)
PMS1-rs1233255						
AA		1		1		1
AC	0.063	0.834(0.689,1.010)	0.824	0.973(0.768,1.233)	0.550	1.083()0.834,1.405
CC	0.232	0.713(0.409,1.242)	0.707	1.162(0.532,2.536)	0.785	0.889(0.382,2.069)
AA/AC+CC	0.040*	0.823(0.684,0.991)	0.902	0.986(0.783,1.241)	0.614	1.067(0.828,1.375)
AA+AC/CC	0.298	0.746(0.429,1.297)	0.694	1.169(0.536,2.547)	0.751	0.873(0.376,2.025)
A/C	0.032*	0.836(0.709,0.985)	0.999	1.000(0.813,1.231)	0.716	0.959(0.763,1.204)
PMS1-rs1233258						
TT		1		1		1
TC	0.303	0.910(0.760,1.089)	0.728	0.962(0.772,1.198)	0.631	1.061(0.834,1.349)
CC	0.893	0.983(0.763,1.265)	0.605	0.924(0.684,1.248)	0.516	1.115(0.802,1.550)
TT/TC+CC	0.381	0.927(0.781,1.099)	0.643	0.952(0.774,1.171)	0.537	1.074(0.856,1.347)

TT+TC/CC	0.760	1.037(0.823,1.306)	0.681	0.944(0.717,1.242)	0.618	1.080(0.799,1.458)
T/C	0.760	1.019(0.904,1.148)	0.677	1.031(0.892,1.192)	0.487	1.058(0.903,1.240)
PMS1-rs5743116						
TT		1		1		1
TC	0.124	0.872(0.733,1.038)	0.523	1.072(0.866,1.328)	0.789	0.968(0.763,1.228)
CC	0.510	1.153(0.755,1.759)	0.072	1.703(0.953,3.045)	0.134	1.457(0.891,2.382)
TT/TC+CC	0.214	0.899(0.760,1.063)	0.276	1.122(0.912,1.381)	0.853	1.022(0.815,1.281)
TT+TC/CC	0.367	1.212(0.798,1.841)	0.084	1.663(0.935,2.959)	0.117	1.473(0.907,2.392)
T/C	0.469	1.053(0.915,1.212)	0.126	0.871(0.729,1.040)	0.562	1.057(0.876,1.275)
PMS2-rs12112229						
CC		1		1		1
CA	0.764	1.031(0.844,1.260)	0.321	0.887(0.701,1.124)	0.042*	1.304(1.010,1.684)
AA	0.481	0.751(0.339,1.664)	0.038*	0.429(0.193,0.952)	0.806	1.154(0.369,3.602)
CC/CA+AA	0.872	1.016(0.835,1.237)	0.17	0.852(0.677,1.071)	0.043*	1.298(1.008,1.670)
CC+CA/AA	0.470	0.746(0.337,1.651)	0.044*	0.441(0.199,0.977)	0.889	1.084(0.348,3.380)
C/A	0.992	1.001(0.836,1.198)	0.084	1.199(0.975,1.474)	0.059	1.248(0.992,1.570)

Abbreviations: NeC, negative control; SC, spontaneous clearance; CHB, chronic hepatitis B; LC, liver cirrhosis; HCC, hepatocellular carcinoma; HLD, HBV-induced liver diseases (CHB+LC+HCC).

Pn: nominal P-values. *: *Pn*＜0.05

Supplementary table7: The negative results of the additive interaction analysis of 11 SNPs in NeC vs. HLD.

SNP1	SNP2	HLD	NeC	B	*P*	OR(95%CI)	RERI/AP/S
rs1540354	rs175080						
TT	AA	2	6			1	
TT	GG+AG	154	79	1.538	0.071	4.655(0.876,24.721)	RERI: -3.747(11.838,4.343)
AA+TA	AA	55	25	1.641	0.062	5.160(0.922,28.869)	AP: -0.739(-1.384,-0.095)
AA+TA	GG+AG	1532	700	1.623	0.054	5.070(0.976,26.342)	S: 0.521(0.360,0.752)
rs1540354	rs1233258						
TT	TT	58	32			1	
TT	TC+CC	98	56	0.281	0.351	1.324(0.734,2.387)	RERI:-0.130(-0.837,0.576)
AA+AT	TT	614	270	0.225	0.354	1.252(0.778,2.016)	AP:-0.090(-0.556,0.376)
AA+AT	TC+CC	999	475	0.369	0.135	1.447(0.891,2.349)	S:0.774(0.267,2.245)
rs1540354	rs12112229						
TT	AA	120	53			1	
TT	CC+CA	36	35	-0.958	0.002	0.384(0.210,0.701)	RERI:0.747(0.467,1.026)
AA+AT	AA	1244	590	-0.195	0.291	0.823(0.573,1.182)	AP:0.783(0.384,1.183)
AA+AT	CC+CA	368	153	-0.048	0.815	0.953(0.637,1.426)	S:0.059(0.000,75.078)
rs175080	rs1150793						
AA	GG	42	27			1	
GG+AG	AG+AA	14	4	0.614	0.339	1.848(0.524,6.518)	RERI:-0.478(-2.674,1.719)
AA	GG	1282	626	0.212	0.445	1.236(0.718,2.129)	AP:-0.297(-1.633,1.039)

GG+AG	AG+AA	408	153	0.474	0.102	1.607(0.910,2.836)	S:0.559(0.079,3.975)
rs175080	rs5742933						
AA	GC+CC	25	19			1	
GG+AG	GG	32	10	0.880	0.082	2.411(0.896,6.488)	RERI:-1.137(-3.290,1.016)
AA	GC+CC	625	320	0.477	0.160	1.611(0.828,3.132)	AP:-0.603(-1.585,0.379)
GG+AG	GG	1059	455	0.634	0.063	1.885(0.967,3.672)	S:0.438(0.181,1.061)
rs175080	rs1233255						
AA	AC+CC	14	13			1	
GG+AG	AA	44	18	0.814	0.117	2.258(0.815,6.255)	RERI:-0.982(-2.976,1.012)
AA	AC+CC	410	221	0.581	0.169	1.787(0.781,4.093)	AP:-0.476(-1.226,0.274)
GG+AG	AA	1286	558	0.724	0.084	2.063(0.907,4.692)	S:0.520(0.265,1.019)
rs175080	rs12112229						
AA	AA	57	32			1	
GG+AG	CC+CA	27	23	-0.952	0.082	0.386(0.132,1.129)	RERI:0.658(0.239,1.077)
AA	AA	1301	610	-0.138	0.647	0.871(0.483,1.571)	AP:0.719(0.017,1.421)
GG+AG	CC+CA	396	169	-0.089	0.775	0.915(0.496,1.686)	S:0.115(0.001,22.747)
rs1150793	rs5742933						
GG	GC+CC	518	282			1	
AG+AA	GG	825	388	0.124	0.299	1.132(0.896,1.430)	RERI:0.441(-0.095,0.978)
GG	GC+CC	142	71	0.091	0.603	1.096(0.776,1.547)	AP:0.264(-0.021,0.550)
AG+AA	GG	282	91	0.512	0.002	1.669(1.215,2.292)	S:2.942(0.427,20.281)
rs1150793	rs1233255						

GG	AC+CC	350	199			1	
AG+AA	AA	974	475	0.130	0.289	1.139(0.896,1.449)	RERI:0.309(-0.252,0.869)
GG	AC+CC	82	40	0.117	0.600	1.125(0.725,1.744)	AP:0.196(-0.148,0.541)
AG+AA	AA	339	124	0.452	0.004	1.572(1.158,2.134)	S:2.173(0.281,16.820)
rs1150793	rs256554						
GG	AA	79	53			1	
AG+AA	CA+CC	1236	616	0.165	0.420	1.180(0.790,1.762)	RERI:-3.100(-7.961,1.761)
GG	AA	24	4	1.478	0.012	4.384(1.393,13.798)	AP:-2.118(-5.341,1.106)
AG+AA	CA+CC	390	158	0.381	0.085	1.463(0.949,2.255)	S:0.130(0.028,0.595)
rs1150793	rs5743116						
GG	TC+CC	493	266			1	
AG+AA	TT	830	405	0.096	0.398	1.101(0.881,1.376)	RERI:-0.038(-0.575,0.498)
GG	TC+CC	172	74	0.303	0.078	1.354(0.967,1.896)	AP:-0.027(-0.409,0.355)
AG+AA	TT	250	90	0.348	0.029	1.417(1.036,1.938)	S:0.915(0.272,3.082)
rs1150793	rs1233258						
GG	TT	487	252			1	
AG+AA	TC+CC	858	419	0.272	0.027	1.313(1.032,1.670)	RERI:-0.744(-1.466,-0.022)
GG	TT	186	50	0.610	0.001	1.840(1.282,2.643)	AP:-0.528(-1.080,0.025)
AG+AA	TC+CC	241	112	0.343	0.032	1.410(1.031,1.928)	S:0.355(0.140,0.901)
rs1150793	rs12112229						
GG	AA	1044	522			1	
AG+AA	CC+CA	300	147	0.022	0.861	1.022(0.803,1.300)	RERI:-0.069(-0.656,0.517)

GG	AA	324	123	0.287	0.023	1.332(1.040,1.706)	AP:-0.054(-0.525,0.417)
AG+AA	CC+CA	104	41	0.251	0.223	1.285(0.858,1.923)	S:0.804(0.120,5.391)
rs5742933	rs1233255						
GC+CC	AC+CC	276	164			1	
GG	AA	377	189	0.136	0.353	1.145(0.860,1.525)	RERI:0.132(-0.306,0.571)
GC+CC	AC+CC	156	73	0.132	0.469	1.141(0.799,1.631)	AP:0.093(-0.220,0.407)
GG	AA	936	406	0.350	0.013	1.419(1.076,1.872)	S:1.461(0.287,7.436)
rs5742933	rs256554						
GC+CC	AA	64	37			1	
GG	CA+CC	576	309	0.094	0.692	1.098(0.691,1.746)	RERI:-0.372(-1.402,0.658)
GC+CC	AA	39	20	0.449	0.223	1.566(0.761,3.223)	AP:-0.288(-1.033,0.457)
GG	CA+CC	1047	457	0.257	0.297	1.293(0.798,2.095)	S:0.440(0.111,1.749)
rs5742933	rs12112229						
GC+CC	AA	507	283			1	
GG	CC+CA	154	67	0.273	0.133	1.314(0.920,1.878)	RERI:-0.489(-1.008,0.031)
GC+CC	AA	860	358	0.271	0.024	1.311(1.037,1.657)	AP:-0.430(-0.915,0.055)
GG	CC+CA	249	121	0.128	0.405	1.137(0.841,1.538)	S:0.218(0.030,1.611)
rs1233255	rs5743116						
AC+CC	TC+CC	159	104			1	
AA	TT	269	132	0.385	0.033	1.469(1.033,2.090)	RERI:-0.530(-1.095,0.035)
AC+CC	TC+CC	501	236	0.406	0.012	1.501(1.093,2.062)	AP:-0.368(-0.725,-0.011)
AA	TT	796	363	0.365	0.033	1.440(1.031,2.012)	S:0.454(0.258,0.799)

rs1233255	rs12112229						
AC+CC	AA	326	195			1	
AA	CC+CA	108	39	0.402	0.069	1.495(0.969,2.307)	RERI:-0.646(-1.321,0.029)
AC+CC	AA	1025	450	0.276	0.025	1.318(1.035,1.680)	AP:-0.554(-1.146,0.038)
AA	CC+CA	294	149	0.154	0.318	1.167(0.862,1.579)	S:0.205(0.039,1.078)
rs256554	rs5743116						
AA	TC+CC	101	51			1	
CA+CC	TT	2	6	-1.763	0.038	0.171(0.032,0.909)	RERI:0.935(0.616,1.254)
AA	TC+CC	549	282	-0.134	0.503	0.875(0.592,1.293)	AP:0.953(0.491,1.415)
CA+CC	TT	1065	487	-0.019	0.921	0.981(0.669,1.438)	S:0.020(0.000,1598528.138)
rs256554	rs1233258						
AA	TT	24	2			1	
CA+CC	TC+CC	79	55	-1.927	0.012	0.146(0.032,0.657)	RERI:0.890(0.722,1.059)
AA	TT	639	298	-1.906	0.011	0.149(0.034,0.647)	AP:4.825(-3.012,12.661)
CA+CC	TC+CC	989	469	-1.69	0.024	0.185(0.043,0.801)	S:0.478(0.438,0.522)
rs256554	rs12112229						
AA	AA	80	43			1	
CA+CC	CC+CA	24	14	-0.050	0.904	0.951(0.421,2.151)	RERI:0.061(-0.681,0.804)
AA	AA	1260	598	-0.028	0.896	0.972(0.639,1.481)	AP:0.062(-0.700,0.825)
CA+CC	CC+CA	368	169	-0.015	0.948	0.985(0.626,1.550)	S:0.195(0.000,224976705.505)
rs5743116	rs12112229						
TC+CC	AA	512	272			1	

TT	CC+CA	157	68	0.181	0.312	1.198(0.844,1.703)	RERI:-0.301(-0.777,0.175)
TC+CC	AA	836	373	0.143	0.204	1.154(0.925,1.439)	AP:-0.286(-0.756,0.183)
TT	CC+CA	242	120	0.050	0.741	1.051(0.782,1.413)	S:0.146(0.001,21.362)
rs1233258	rs12112229						
TT	AA	524	227			1	
TC+CC	CC+CA	152	75	-0.063	0.714	0.939(0.669,1.317)	RERI:0.124(-0.297,0.546)
TT	AA	847	416	0.130	0.296	1.139(0.893,1.452)	AP:0.103(-0.238,0.445)
TC+CC	CC+CA	255	113	0.184	0.251	1.203(0.878,1.647)	S:2.598(0.016,434.120)

Abbreviations: RERI, Relative Excess Risk of Interaction; AP, Attributable Proportion of interaction; S: Synergy index.

When calculating covariance matrix, take SNPs other than the analysis SNPs, together with gender, age, the history of drinking and smoking as control variables. The bold font shows statistical significance.

Supplementary table8: The negative results of the additive interaction analysis of 11 SNPs in SC vs. HLD.

SNP1	SNP2	HLD	SC	B	*P*	OR(95%CI)	RERI/AP/S
rs1540354	rs175080						
AA+AT	GG	1060	300			1	
AA+AT	AG+AA	527	138	0.078	0.518	1.081(0.853,1.370)	RERI:0.276(-1.905,2.456)
TT	GG	110	16	0.643	0.023	1.902(1.093,3.309)	AP:0.122(-0.753,0.998)
TT	AG+AA	46	6	0.815	0.067	2.260(0.946,5.400)	S:1.281(0.201,8.178)
rs1540354	rs5742933						
AA+AT	CC	608	186			1	
AA+AT	GG+GC	1010	286	0.125	0.369	1.133(0.863,1.487)	RERI:1.255(-0.693,3.203)
TT	CC	61	12	0.383	0.256	1.467(0.757,2.843)	AP:0.440(-0.031,0.910)
TT	GG+GC	95	12	1.049	0.003	2.856(1.415,5.765)	S:3.092(0.485,19.711)
rs1540354	rs256554						
AA+AT	CC	811	302			1	
AA+AT	CA+AA	725	166	0.807	0.001	2.240(1.418,3.540)	RERI:-0.471(-3.036,2.094)
TT	CC	94	14	0.881	0.006	2.414(1.291,4.516)	AP:-0.148(-1.037,0.741)
TT	CA+AA	62	10	1.158	0.005	3.184(1.418,7.148)	S:0823(0.270,2.504)
rs1540354	rs1233258						
AA+AT	CC	247	82			1	
AA+AT	TT+TC	1371	390	0.314	0.068	1.369(0.977,1.918)	RERI:0.381(-2.031,2.794)

TT	CC	25	4	0.659	0.249	1.933(0.630,5.929)	AP:0.142(-0.727,1.011)
TT	TT+TC	131	20	0.987	0.001	2.682(1.483,4.850)	S:1.293(0.223,7.508)
rs175080	rs5742933						
GG	CC	440	146			1	
AG+AA	GG+GC	767	204	0.236	0.127	1.266(0.935,1.714)	RERI:-0.290(-0.852,0.271)
GG	CC	237	56	0.241	0.208	1.273(0.874,1.853)	AP:-0.232(-0.696,0.232)
AG+AA	GG+GC	338	90	0.222	0.215	1.249(0.879,1.774)	S:0.461(0.114,1.871)
rs175080	rs256554						
GG	CC	678	232			1	
AG+AA	CA+AA	531	118	0.959	0.000	2.610(1.611,4.230)	RERI:-0.958(-1.833,-0.083)
GG	CC	337	82	0.305	0.045	1.357(1.007,1.829)	AP:-0.477(-0.991,0.037)
AG+AA	CA+AA	238	64	0.697	0.010	2.008(1.183,3.407)	S:0.513(0.272,0.965)
rs175080	rs1233258						
GG	CC	203	74			1	
AG+AA	TT+TC	1006	276	0.22	0.263	1.247(0.847,1.834)	RERI:0.331(-0.198,0.861)
GG	CC	80	28	-0.168	0.559	0.845(0.481,1.485)	AP:0.233(-0.141,0.607)
AG+AA	TT+TC	495	118	0.352	0.095	1.422(0.940,2.151)	S:4.658(0.005,4529.896)
rs175080	rs5743116						
GG	TT	742	236			1	
AG+AA	TC+CC	467	114	-0.356	0.163	0.701(0.425,1.155)	RERI:-0.361(-0.778,0.056)

GG	TT	347	84	0.23	0.126	1.258(0.937,1.688)	AP:-0.604(-1.347,0.140)
AG+AA	TC+CC	228	62	-0.514	0.056	0.598(0.353,1.014)	S:9.280(0.000,384998.567)
rs1150793	rs1233258						
AA+AG	CC	271	82			1	
GG	TT+TC	1478	396	0.301	0.076	1.351(0.969,1.883)	RERI:1.557(-1.183,4.297)
AA+AG	CC	8	2	-0.187	0.832	0.830(0.148,4.660)	AP:0569(-0.044,1.1820)
GG	TT+TC	28	6	1.007	0.077	2.736(0.897,8.341)	S:9.617(0.004,22174.467)
rs5742933	rs256554						
CC	CC	202	78			1	
GG+GC	CA+AA	494	122	0.944	0.001	2.569(1.452,4.546)	RERI:-0.246(-1.219,0.727)
CC	CC	811	240	0.259	0.135	1.295(0.923,1.818)	AP:-0.094(-0.479,0.291)
GG+GC	CA+AA	278	56	0.963	0.000	2.619(1.560,4.399)	S:0.868(0.499,1.511)
rs5742933	rs1233258						
CC	CC	212	62			1	
GG+GC	TT+TC	464	138	0.222	0.260	1.249(0.848,1.839)	RERI:0.385(-0.174,0.944)
CC	CC	65	22	-0.101	0.746	0.904(0.492,1.662)	AP:0.250(-0.133,0.633)
GG+GC	TT+TC	1046	274	0.43	0.036	1.537(1.028,2.299)	S:3.523(0.036,340.378)
rs5742933	rs12112229						
CC	AA	16	6			1	
GG+GC	CC+CA	660	194	0.901	0.171	2.462(0.677,8.948)	RERI:-0.107(-2.032,1.818)

CC	AA	10	6	0.078	0.927	1.082(0.200,5.859)	AP:-0.055(-1.080,0.970)
GG+GC	CC+CA	1101	290	1.059	0.109	2.884(0.791,10.521)	S:0.899(0.126,6.420)
rs256554	rs1233255						
CC	AA+AC	969	312			1	
CA+AA	CC	36	4	-0.094	0.884	0.910(0.255,3.244)	RERI:0.844(-1.471,3.159)
CC	AA+AC	719	174	0.765	0.001	2.149(1.363,3.389)	AP:0.352(-0.375,1.080)
CA+AA	CC	64	6	1.17	0.051	3.221(0.996,10.420)	S:2.533(0.194,33.011)
rs256554	rs1233258						
CC	CC	44	22			1	
CA+AA	TT+TC	970	294	0.446	0.179	1.562(0.815,2.993)	RERI:0.244(-0.826,1.314)
CC	CC	221	62	0.931	0.023	2.537(1.137,5.662)	AP:0.073(-0.254,0.400)
CA+AA	TT+TC	553	118	1.207	0.002	3.344(1.548,7.222)	S:1.116(0.658,1.893)
rs256554	rs5743116						
CC	TT	948	306			1	
CA+AA	TC+CC	65	10	0.054	0.898	1.055(0.462,2.413)	RERI:-1.727(-3.670,0.216)
CC	TT	124	12	1.088	0.001	2.969(1.595,5.529)	AP:-1.332(-2.856,0.193)
CA+AA	TC+CC	651	168	0.26	0.069	1.297(0.980,1.718)	S:0.147(0.042,0.511)
rs256554	rs12112229						
CC	AA	11	8			1	
CA+AA	CC+CA	1002	308	1.219	0.020	3.403(1.232,9.400)	RERI:-0.206(-8.142,7.729)

CC	AA	10	2	1.631	0.100	3.498(0.523,23.378)	AP:-0.028(-1.115,1.058)
CA+AA	CC+CA	165	178	1.986	0.000	4.958(1.771,13.878)	S:0.968(0.286,3.276)
rs5743116	rs12112229						
TT	AA	11	8			1	
TC+CC	CC+CA	1096	312	1.43	0.008	4.179(1.459,11.972)	RERI:-3.154(-8.510,2.203)
TT	AA	12	2	0.914	0.361	2.495(0.350,17.780)	AP:-1.252(-2.834,0.331)
TC+CC	CC+CA	669	174	0.924	0.115	2.519(0.799,7.938)	S:0.325(0.135,0.781)

Abbreviations: RERI, Relative Excess Risk of Interaction; AP, Attributable Proportion of interaction; S: Synergy index. When calculating covariance matrix, take SNPs other than the analysis SNPs, together with gender, age, the history of drinking and smoking as control variables. The bold font shows statistical significance.

Supplementary table9: The multivariate Logistic Regression analysis of predictive factors for liver cancerization after HBV infection under three models in CHB+LC vs. HCC group.

Variable	B	S.E	Wald	P_n	OR (95%CI)
Codominant					
age	0.984	0.100	96.115	0.000*	2.674(2.197,3.255)
sex (Male)	0.326	0.156	4.404	0.036*	1.386(1.022,1.880)
smoke (Yes)	0.629	0.134	22.149	0.000*	1.876(1.443,2.437)
Dominant					
age	0.984	0.100	96.115	0.000*	2.674(2.197,3.255)
sex (Male)	0.326	0.156	4.404	0.036*	1.386(1.022,1.880)
smoke (Yes)	0.629	0.134	22.149	0.000*	1.876(1.443,2.437)
Recessive					
age	0.991	0.101	96.798	0.000*	2.693(2.211,3.280)
sex (Male)	0.331	0.156	4.508	0.034*	1.392(1.026,1.890)
smoke (Yes)	0.619	0.134	21.382	0.000*	1.858(1.429,2.415)
PMS1-rs5743116(CC)	0.526	0.269	3.824	0.051	1.691(0.999,2.864)

Abbreviations: CHB, chronic hepatitis B; LC, liver cirrhosis; HCC, hepatocellular carcinoma. *Pn*: nominal P-values. *: *Pn* < 0.05.

Supplementary table10: The negative results of the additive interaction analysis of 11 SNPs in CHB+LC vs. HCC.

SNP1	SNP2	HCC	CHB+L	B	*P*	OR(95%CI)	RERI/AP/S
rs1540354	rs175080						
TA+TT	AG+GG	197	690			1	
TA+TT	AA	6	17	0.402	0.435	1.496(0.545,4.107)	RERI:0.059(-1.797,1.915)
AA	AG+GG	196	603	0.122	0.327	1.130(0.885,1.444)	AP:0.035(-1.051,1.122)
AA	AA	11	23	0.521	0.225	1.685(0.725,3.913)	S:1.095(0.062,19.283)
rs1540354	rs1150793						
TA+TT	AA	157	559			1	
TA+TT	AG+GG	49	163	0.040	0.844	1.041(0.698,1.551)	RERI:0.014(-0.553,0.580)
AA	AA	159	471	0.121	0.392	1.128(0.856,1.487)	AP:0.011(-0.466,0.488)
AA	AG+GG	53	159	0.168	0.403	1.183(0.798,1.752)	S:1.080(0.041,28.431)
rs1540354	rs5742933						
TA+TT	GC+GG	189	696			1	
TA+TT	CC	15	24	0.636	0.112	1.890(0.862,4.141)	RERI:-0.390(-2.067,1.287)
AA	GC+GG	200	601	0.138	0.276	1.147(0.896,1.470)	AP:-0.237(-1.364,0.891)
AA	CC	12	27	0.499	0.231	1.646(0.729,3.719)	S:0.624(0.077,5.079)
rs1540354	rs1233255						
TA+TT	AA	144	542			1	
TA+TT	AC+CC	57	171	0.309	0.134	1.362(0.909,2.041)	RERI:-0.522(-1.171,0.126)
AA	AA	160	463	0.237	0.097	1.267(0.958,1.676)	AP:-0.471(-1.125,0.182)
AA	AC+CC	49	156	0.102	0.631	1.107(0.731,1.675)	S:0.171(0.005,6.166)
rs1540354	rs256554						

TA+TT	CC	106	397			1	
TA+TT	CA+AA	93	312	-	0.629	0.908(0.615,1.341)	RERI:0.008(-0.444,0.461)
AA	CC	119	364	0.114	0.483	1.121(0.815,1.543)	AP:0.008(-0.428,0.444)
AA	CA+AA	86	256	0.037	0.857	1.037(0.696,1.547)	S:1.290(0.000,6654171.260)
rs1540354	rs5743116						
TA+TT	TC+TT	191	685			1	
TA+TT	CC	11	28	-	0.855	0.924(0.397,2.151)	RERI:0.136(-0.883,1.155)
AA	TC+TT	196	592	0.116	0.358	1.123(0.877,1.437)	AP:0.115(-0.696,0.926)
AA	CC	14	28	0.168	0.688	1.182(0.521,2.682)	S:3.889(0.000,32526277.437)
rs1540354	rs1233258						
TA+TT	TT	76	266			1	
TA+TT	TC+CC	130	456	-	0.869	0.966(0.643,1.453)	RERI:0.244(-0.192,0.680)
AA	TT	76	254	-	0.917	0.979(0.662,1.449)	AP:0.205(-0.169,0.579)
AA	TC+CC	135	376	0.174	0.399	1.190(0.794,1.784)	S:-3.506(#NUM!,#NUM!)
rs175080	rs1150793						
AG+GG	AA	298	984			1	
AA	AG+GG	96	312	0.024	0.868	1.025(0.769,1.365)	RERI:0.784(-1.619,3.187)
AG+GG	AA	11	31	0.253	0.537	1.288(0.577,2.875)	AP:0.374(-0.423,1.171)
AA	AG+GG	6	8	0.740	0.199	2.095(0.677,6.482)	S:3.511(0.092,133.796)
rs175080	rs1233255						
AG+GG	AA	287	970			1	
AA	AC+CC	101	309	0.089	0.570	1.093(0.804,1.486)	RERI:0.003(-2.127,2.133)

AG+GG	AA	12	29	0.408	0.290	1.503(0.707,3.197)	AP:0.002(-1.327,1.331)
AA	AC+CC	4	10	0.470	0.463	1.600(0.456,5.608)	S:1.005(0.029,35.162)
rs175080	rs1233258						
AG+GG	TT	143	496			1	
AA	TC+CC	250	800	0.093	0.573	1.098(0.793,1.520)	RERI:-0.418(-2.325,1.489)
AG+GG	TT	7	18	0.570	0.257	1.768(0.660,4.736)	AP:-0.289(-1.734,1.156)
AA	TC+CC	10	22	0.370	0.419	1.448(0.590,3.551)	S:0.517(0.026,10.437)
rs1150793	rs5742933						
AA	GC+GG	297	988			1	
AG+GG	CC	18	40	0.506	0.142	1.659(0.843,3.265)	RERI:0.007(-1.782,1.796)
AA	GC+GG	94	310	0.044	0.763	1.045(0.783,1.395)	AP:0.004(-1.038,1.047)
AG+GG	CC	9	11	0.537	0.322	1.711(0.591,4.953)	S:1.010(0.081,12.616)
rs1150793	rs1233258						
AA	TT	112	377			1	
AG+GG	TC+CC	204	654	0.059	0.744	1.060(0.745,1.510)	RERI:0.100(-0.436,0.637)
AA	TT	43	114	-	0.962	0.989(0.636,1.539)	AP:0.087(-0.371,0.546)
AG+GG	TC+CC	61	180	0.14	0.535	1.150(0.740,1.788)	S:3.015(0.000,121563.902)
rs5742933	rs256554						
GC+GG	CC	234	779			1	
CC	CA+AA	157	522	-	0.712	0.943(0.690,1.289)	RERI:-7.966(-4.996,1.063)
GC+GG	CC	8	15	1.222	0.104	3.393(0.779,14.771)	AP:-1.435(-3.905,1.035)
CC	CA+AA	21	46	0.315	0.369	1.371(0.689,2.727)	S:0.158(0.016,1.571)

rs5742933	rs1233255						
GC+GG	AA	300	1008			1	
CC	AC+CC	92	302	0.066	0.679	1.068(0.781,1.462)	RERI:0.573(-0.932,2.078)
GC+GG	AA	12	24	0.265	0.631	1.303(0.442,3.840)	AP:0.295(-0.353,0.942)
CC	AC+CC	16	31	0.665	0.053	1.944(0.992,3.811)	S:2.541(0.145,44.660)
rs5742933	rs1233258						
GC+GG	TT	154	527			1	
CC	TC+CC	238	784	0.082	0.615	1.086(0.787,1.497)	RERI:-0.751(-4.863,3.362)
GC+GG	TT	1	3	0.585	0.089	1.795(0.916,3.521)	AP:-0.664(-4.351,3.022)
CC	TC+CC	27	51	0.122	0.319	1.130(0.889,1.437)	S:0.147(0.000,75.013)
rs5742933	rs5743116						
GC+GG	TC+TT	377	1264			1	
CC	CC	15	47	-	0.694	0.855(0.391,1.868)	RERI:0.582(-1.058,2.222)
GC+GG	TC+TT	14	33	0.369	0.326	1.446(0.692,3.018)	AP:0.309(-0.413,1.032)
CC	CC	14	21	0.633	0.118	1.884(0.851,4.170)	S:2.934(0.068,127.107)
rs256554	rs1233258						
CC	TT	157	511			1	
CA+AA	TC+CC	83	284	0.053	0.77	1.054(0.741,1.500)	RERI:0.165(-0.465,0.794)
CC	TT	11	34	-0.26	0.572	0.771(0.313,1.899)	AP:0.166(-0.470,0.802)
CA+AA	TC+CC	170	538	-0.01	0.95	0.990(0.729,1.345)	S:0.057(0.000,1062470699.908)
rs1233255	rs1233258						
AA	TT	148	504			1	

AC+CC	TC+CC	160	522	0.077	0.664	1.080(0.764,1.526)	RERI:0.057(-0.701,0.815)
AA	TT	13	36	0.046	0.928	1.047(0.387,2.833)	AP:0.048(-0.591,0.687)
AC+CC	TC+CC	100	305	0.169	0.377	1.184(0.814,1.723)	S:1.448(0.004,540.118)
rs1233255	rs5743116						
AA	TC+TT	291	983			1	
AC+CC	CC	18	44	-	0.962	0.982(0.460,2.096)	RERI:0.016(-1.080,1.111)
AA	TC+TT	103	326	0.087	0.591	1.091(0.795,1.496)	AP:0.014(-0.982,1.011)
AC+CC	CC	9	14	0.085	0.868	1.089(0.398,2.984)	S:1.214(0.000,697115.935)

Abbreviations: RERI, Relative Excess Risk of Interaction; AP, Attributable Proportion of interaction; S: Synergy index.

When calculating covariance matrix, take SNPs other than the analysis SNPs, together with gender, age, the history of drinking and smoking as control variables. The bold font shows statistical significance.

1.2 Supplementary Figures

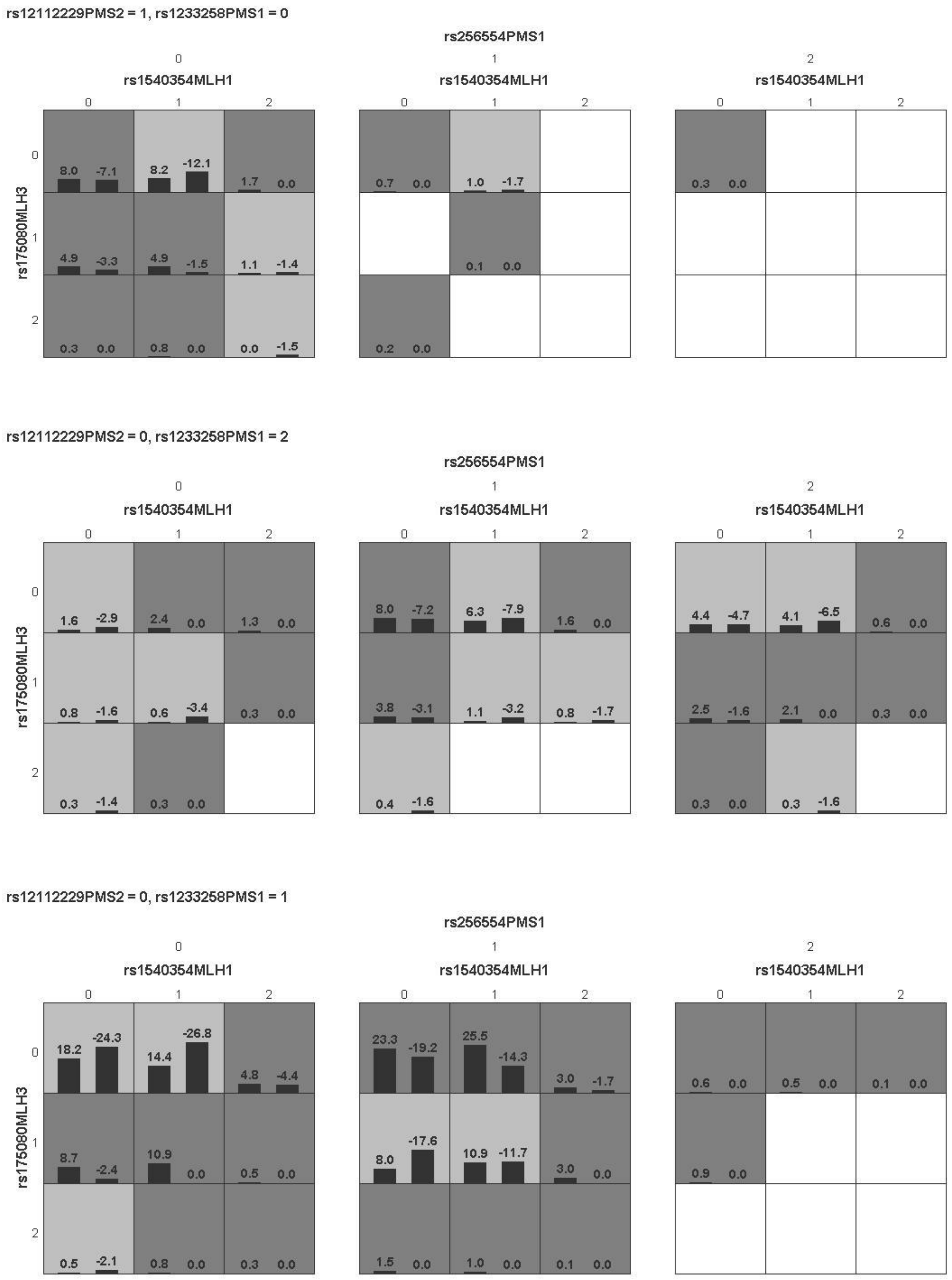

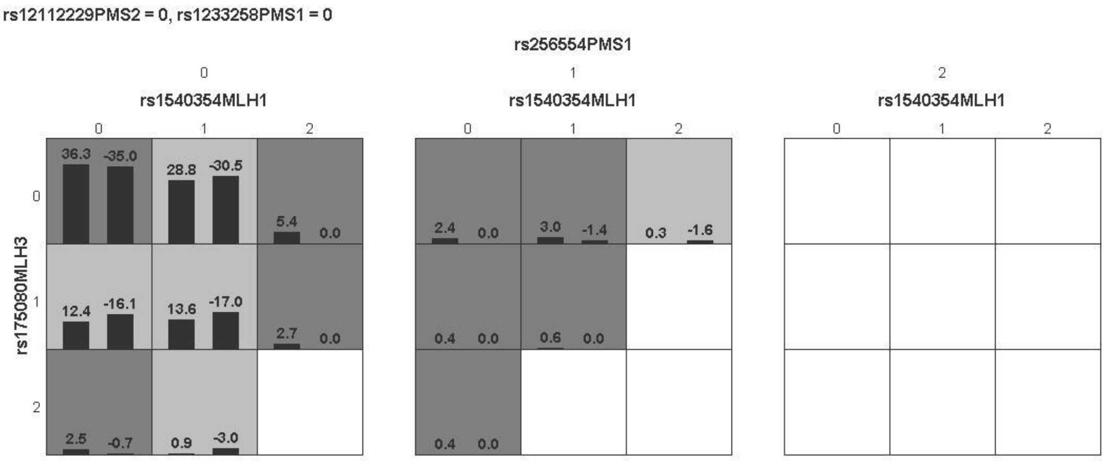
rs12112229PMS2 = 0, rs1233258PMS1 = 0
rs256554PMS1
rs1540354MLH1
rs175080MLH3
36.3 -35.0
28.8 -30.5
5.4 0.0
2.4 0.0
3.0 -1.4
0.3 -1.6
12.4 -16.1
13.6 -17.0
2.7 0.0
0.4 0.0
0.6 0.0
2.5 -0.7
0.9 -3.0
0.4 0.0

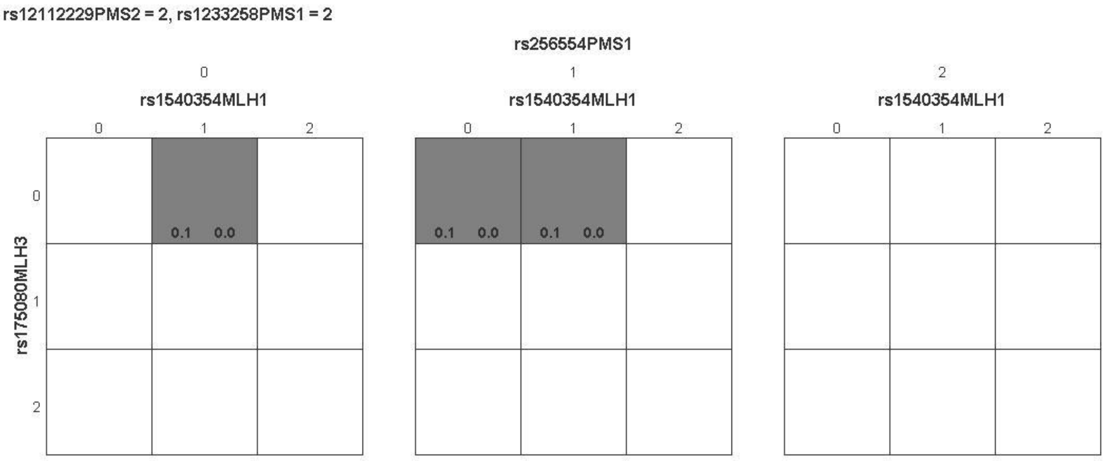
rs12112229PMS2 = 2, rs1233258PMS1 = 2
rs256554PMS1
rs1540354MLH1
rs175080MLH3
0.1 0.0
0.1 0.0
0.1 0.0

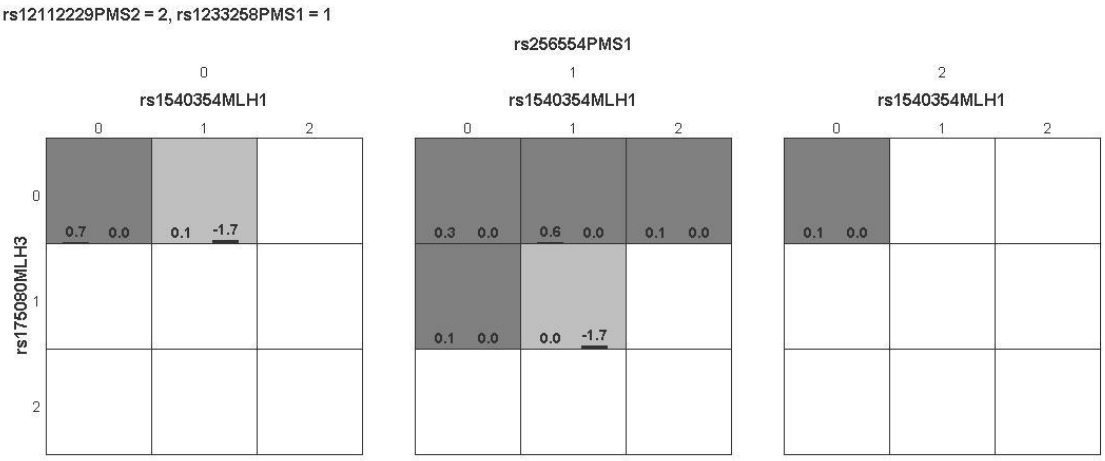
rs12112229PMS2 = 2, rs1233258PMS1 = 1
rs256554PMS1
rs1540354MLH1
rs175080MLH3
0.7 0.0
0.1 -1.7
0.3 0.0
0.6 0.0
0.1 0.0
0.1 0.0
0.1 0.0
0.0 -1.7

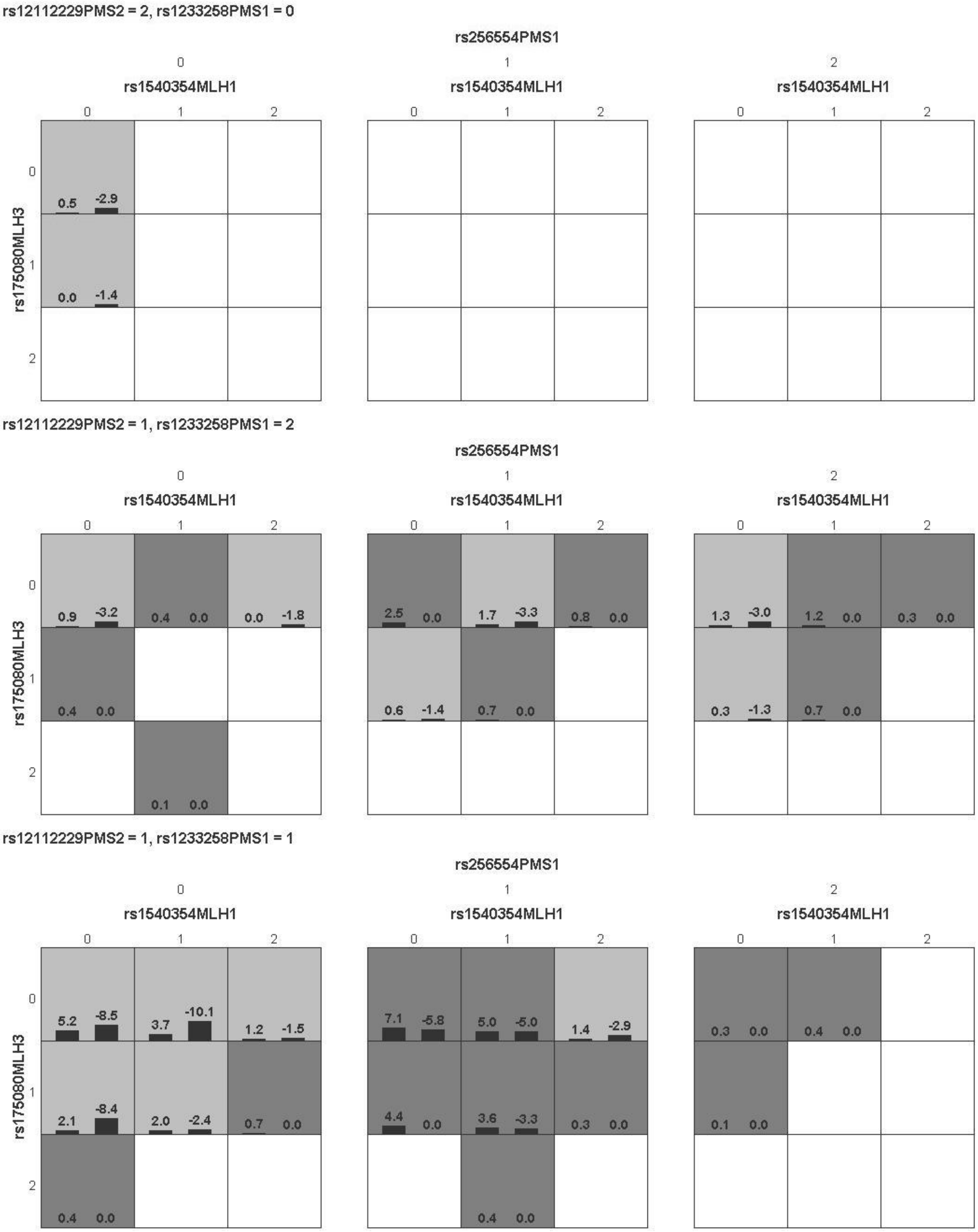

Supplementary Figure 1. The interaction pattern diagram of 5 loci (MLH1-rs1540354A>T, *MLH3*-rs175080G>A, *PMS1*-rs256554 C>A, *PMS1*-1233258T>C and *PMS2*-rs12112229C>A) affecting the ability to clear HBV spontaneously
